# An Arabidopsis mutant deficient in phosphatidylinositol-4-phosphate kinases ß1 and ß2 displays altered auxin-related responses in roots

**DOI:** 10.1038/s41598-022-10458-8

**Published:** 2022-04-28

**Authors:** Anastasiia Starodubtseva, Tetiana Kalachova, Katarzyna Retzer, Adriana Jelínková, Petre Dobrev, Jozef Lacek, Romana Pospíchalová, Jindřiška Angelini, Anne Guivarc’h, Stéphanie Pateyron, Ludivine Soubigou-Taconnat, Lenka Burketová, Eric Ruelland

**Affiliations:** 1 Institute of Experimental Botany of the Czech Academy of Sciences, Rozvojová 263, 165 02, Prague, Czech Republic; 2 University of Chemistry and Technology, Technická 5, 16628 Prague, Czech Republic; 3 Sorbonne Université, UPEC, CNRS, IRD, INRAE, Institute of Ecology and Environmental Sciences of Paris (iEES), 75005 Paris, France; 4 Université de Paris, CNRS, INRAE, Institute of Plant Sciences Paris-Saclay (IPS2), 91405 Orsay, France; 5 Université Paris-Saclay, CNRS, INRAE, Univ Evry, Institute of Plant Sciences Paris-Saclay (IPS2), 91405 Orsay, France; 6 Université de Technologie de Compiègne, Enzyme and Cell Engineering Laboratory, CNRS, 60203 Compiègne, France

**Keywords:** Plant cell biology, Plant signalling

## Abstract

Phosphatidylinositol 4-kinases (PI4Ks) are the first enzymes that commit phosphatidylinositol into the phosphoinositide pathway. Here, we show that *Arabidopsis thaliana* seedlings deficient in PI4Kβ1 and β2 have several developmental defects including shorter roots and unfinished cytokinesis. The *pi4kβ1β2* double mutant was insensitive to exogenous auxin concerning inhibition of root length and cell elongation; it also responded more slowly to gravistimulation. The *pi4kß1ß2* root transcriptome displayed some similarities to a wild type plant response to auxin. Yet, not all the genes displayed such a constitutive auxin-like response. Besides, most assessed genes did not respond to exogenous auxin. This is consistent with data with the transcriptional reporter DR5-GUS. The content of bioactive auxin in the *pi4kß1ß2* roots was similar to that in wild-type ones. Yet, an enhanced auxin-conjugating activity was detected and the auxin level reporter DII-VENUS did not respond to exogenous auxin in *pi4kß1ß2* mutant*.* The mutant exhibited altered subcellular trafficking behavior including the trapping of PIN-FORMED 2 protein in rapidly moving vesicles. Bigger and less fragmented vacuoles were observed in *pi4kß1ß2* roots when compared to the wild type. Furthermore, the actin filament web of the *pi4kß1ß2* double mutant was less dense than in wild-type seedling roots, and less prone to rebuilding after treatment with latrunculin B. A mechanistic model is proposed in which an altered PI4K activity leads to actin filament disorganization, changes in vesicle trafficking, and altered auxin homeostasis and response resulting in a pleiotropic root phenotypes.

## Introduction

Plant health and productivity depends on root outgrowth, which allows water and nutrient uptake, and is equally crucial for an efficient photosynthesis rate^1,2^. Root morphogenesis is a complex process, orchestrated by a complex signaling crosstalk at different levels, from single-cell metabolism to hormone transport within plant organs. On-point spatial and temporal organization of cell organelles, polar establishment of cell architecture and directed shoot ward auxin transport are fundamental for correct root cell differentiation. Root hair cell priming and plasticity require fine-tuned, interconnected cellular processes driven by a properly established cytoskeleton that controls the polar delivery of membranes to the root apex in order to enlarge the cell unidirectionally, and by the transport of auxin through the root tip^1,2^. Auxin regulates cell polarity through the activation of ROPs (Rho-like GTPase) that participate in the polar localization of PIN-FORMED (PIN) family proteins. Carriers of the PIN family are plasma membrane-integrated auxin efflux carriers responsible for the direction and intensity of auxin flow through the plant body. Their cellular localization and activity are regulated at many levels^3–5^, and rely on the lipid composition of the membrane they are in.

Phosphoinositides, minor components of plasma membrane, are phosphorylated derivatives of phosphatidylinositol (PI), such as phosphatidylinositol-4-phosphate (PI4P) and phosphatidylinositol-4,5-bisphosphate (PI4,5P2). Phosphoinositides are important signaling molecules as they are substrates or cofactors of important signaling enzymes. In plants, both PI4P and PI4,5P2 can be substrates to phospholipases C (PLCs) leading to diacylglycerol and the corresponding phosphorylated inositol. PI4,5P2 is a cofactor of some phospholipases D (PLDs), that catalyze the production of phosphatidic acid, a major plant signaling lipid^6^. More generally, phosphoinositides can directly interact with membrane proteins (such as ion channels or G protein-coupled receptors) or cytosolic proteins that they can recruit to membranes^7,8^. Interestingly, specific relative levels of phosphoinositides are a characteristic feature of different membranes: plasma membrane, endoplasmic reticulum and Golgi membranes do not have the same relative composition in phosphoinositides^7–9^. Besides, membrane nanoclusters enriched in certain proteins crucial for signal transduction and transport proteins also have a specific composition in phosphoinositides^8,10^. Formation of membrane domains enriched in PI4P and PI4,5P2 is a crucial component of plasma membrane dynamics. Such phosphoinositide-enriched domains are important for the localization of REMORINs, scaffold proteins governing PM–bound signaling^11^, and FLS2, a pattern-recognition receptor that determines the specific perception of the bacterial protein flagellin^12^.

Composition in phosphoinositides is modified by the activities of lipid kinases. PI4Ks phosphorylate the 4th hydroxyl position in the inositol head group of PI to generate PI4P. PI4P can be further phosphorylated by phosphatidylinositol-4,5-kinases (PI4,5 K) into PI4,5P2. There are two types of PI4Ks according to their primary sequences and pharmacological sensitivities. Type-II PI4Ks are inhibited by adenosine while type III PI4Ks are inhibited by micromolar concentrations of wortmannin, a steroid produced by the fungi *Penicillium funiculosum*. In the *A. thaliana* genome, twelve putative PI4K isoforms have been identified. Eight belong to type-II (*At*PI4Kγ1-8), and four belong to type-III (*At*PI4Kα1 and α2 and *At*PI4Kβ1 and β2)^13^. Not much is known about type-II PI4Ks and they could actually be protein kinases and not lipid kinases^13,14^. We have previously shown that type-III PI4Ks are upstream of the PLC activity that controls the responses of tobacco BY2 cells to cryptogein, a fungi elicitor^15^. Type-III PI4Ks are also upstream of plant cold response PLC activity^16^ but also of the PLC activity that controls gene expression, in basal, non-stimulated conditions^17^. Type-III PI4Ks have been shown also to be activated in response to salicylic acid (SA), and the consequent increase in phosphoinositides is an important part of the specific response of Arabidopsis to this phytohormone^18–20^. *AtPI4Kα2* is a pseudogene and viable homozygous *PI4Kα1* mutants have never been obtained. We have worked previously on a double mutant defective in both *PI4Kβ* genes. In 4-week-old plants, *pi4kβ1β2* exhibited a constitutively high SA level that led to a stunted phenotype^21^. However, SA accumulation did not occur in young *pi4kβ1β2* seedlings^21,22^ and therefore, they appeared to be the material of choice to study the roles of PI4Ks and phosphoinositides in root development. Several aspects of the role of PI4Ks in plant cell biology have been discovered using *pi4kß1ß2* double mutant, such as the involvement of PI4K*β*1 in cell plate formation during cytokinesis^23^, in the formation of secretory vesicles^24^, root hair shaping and polar growth^25^. Here, we show that the *pi4kß1ß2* double mutant exhibits several root phenotypes: impaired primary root growth, lower sensitivity to exogenous indole-3-acetic acid (IAA), impaired elongation and bending in response to gravistimulation, and misshapen root hair growth. These changes appeared to coincide with an altered subcellular distribution and turnover of PIN2, a less stable actin cytoskeleton and generally altered intracellular trafficking dynamics. Moreover, expression of several auxin-associated genes in roots was not responsive to exogenous auxin. However, some transcript levels in non-treated mutant roots were already at the auxin-response levels of wild type (WT) roots, thus displaying an apparent auxin-like response. Remarkably, the measured content of bioactive IAA in double mutant roots did not differ from that of WT, but a considerable increase of glutamate-conjugated form of auxin was monitored. Besides, the auxin level reporter DII-VENUS did not respond to exogenous auxin in *pi4kß1ß2* mutant*.* Our data, therefore, link altered PI4K activity to the modification of vesicular trafficking and actin filaments organization one the one hand to altered auxin response likely due to alteration in auxin homeostasis one the other hand.

## Results

### The* pi4kß1ß2* mutant is impaired in root growth

**Figure 1:**
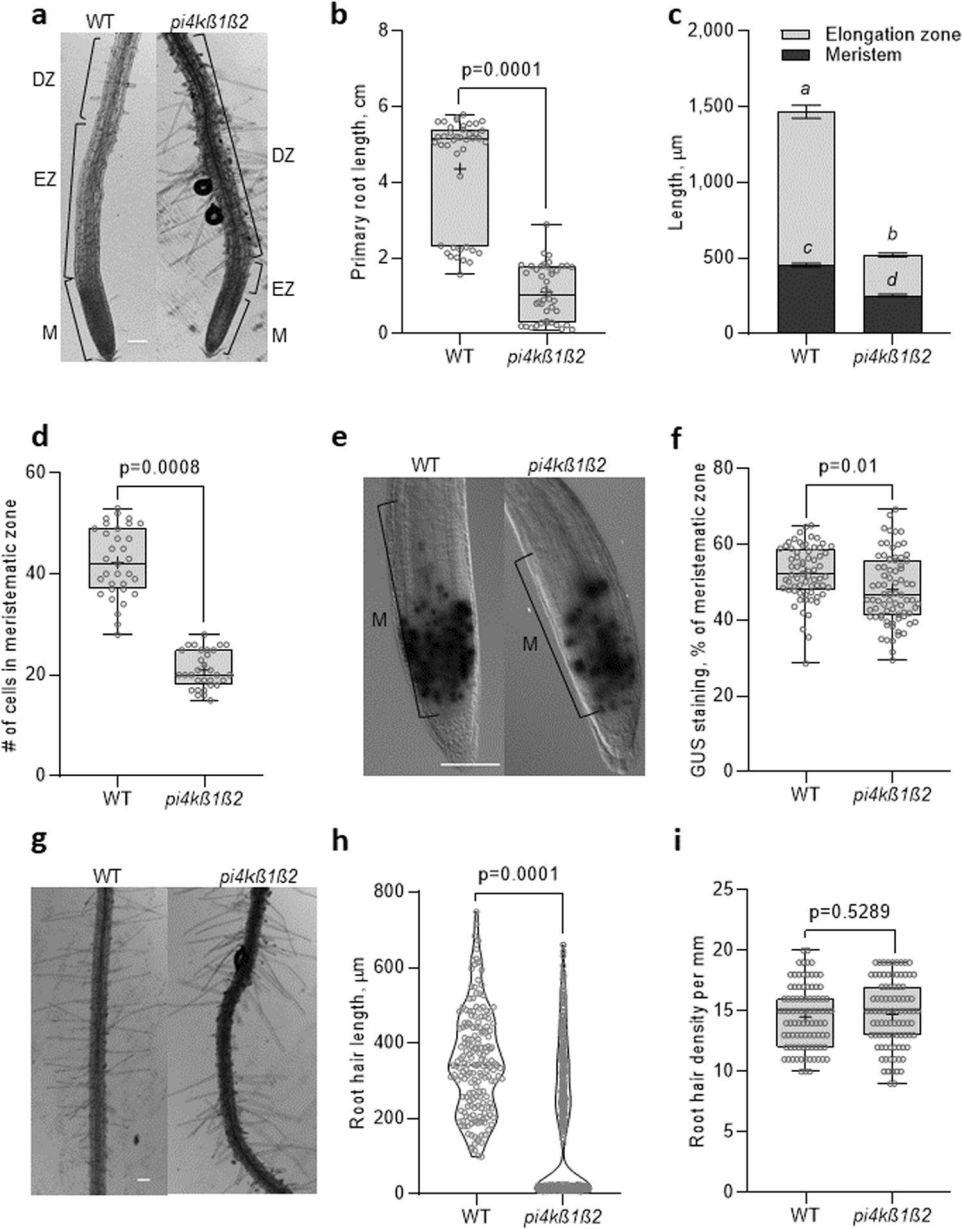
Impaired root growth and morphological characteristics of the *pi4kß1ß2* mutant. (**a**) representative pictures of the apical root parts of 11-day-old seedlings of *Arabidopsis thaliana* Col-0 (WT) and the *pi4kß1ß2* mutant: meristem (M), elongation zone (EZ) and differentiation zone (DZ) are marked, scale bar = 100 μm; (**b**) primary root length, n = 40; (**c**) length of the meristematic and elongation zones, n = 12, error bars represent mean ± SEM; different letters indicate statistically significant groups, one-way ANOVA with Tukey-HSD post-hoc test (p > 0.05); (**d**) number of separated cells in the meristem, n = 36; (**e**) representative images of GUS staining in the root meristem of 4-day-old plants expressing CycB1::GUS, scale bar = 100 μm; (**f**) relative area of CycB1::GUS expression, % of the meristematic zone; n = 72; (**g**) representative images of root hair distribution in the DZ of roots, scale bar = 100 μm; (**h**) root hair length, n = 180; (**i**) root hair density, n = 90. Central line of the boxplots represents the median, plus represents the mean, circles represent individual values from three biological repeats. *p*-value was calculated by Student t-test.

The PI4Kß1ß2 deficiency in *pi4kß1ß2* seedlings led to a decreased primary root length of up to fourfold compared to the WT control (Fig. 1a,b). The shorter primary root of the mutant appeared to be due to shorter meristem and elongation zones (Fig. 1c). The shorter meristem of *pi4kß1ß2* was due to fewer cells (Fig. 1d), some of which showed unfinished cytokinesis. Interestingly, the CycB1::GUS associated signal occupied a smaller percentage area of the meristem in *pi4kß1ß2* roots when compared to the WT (Fig. 1e,f). The elongation zone was almost missing. In the differentiation zone, the *pi4kß1ß2* mutant had smaller cortical cells (Supplementary Fig. S1) and either similar or very small root hair lengths when compared to the WT. This created apparent “bald-like” zones (Fig. 1g,h), while the overall total root hair density in *pi4kß1ß2* plants did not differ to that of the WT (Fig. 1i). An analysis of the epidermal cell lines^26^ showed that the regularity of trichoblasts/atrichoblasts formation was not affected in the mutant (Supplementary Fig. S2). This confirmed that the apparent "bald-like zones'' were not due to an absence of hairs but to shorter root hairs.

### Responses to IAA and to gravistimulation are impaired in *pi4kß1ß2*

**Figure 2:**
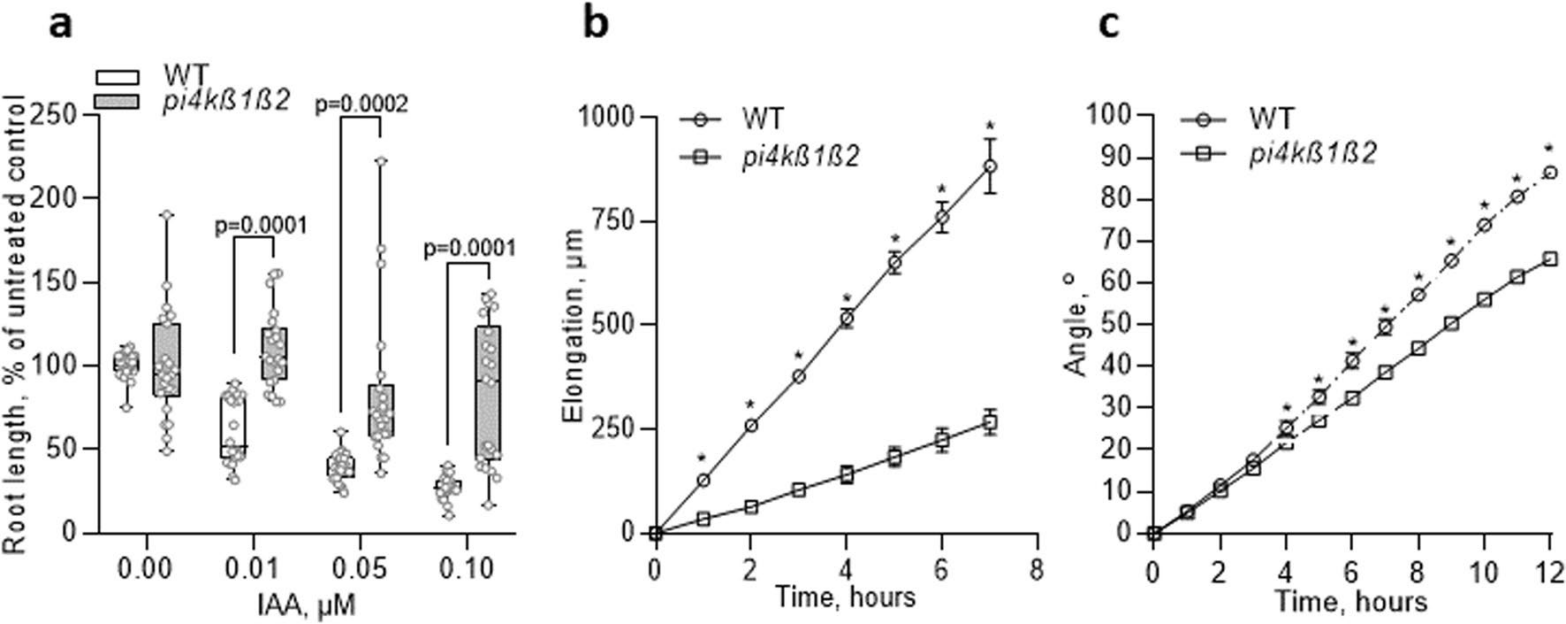
Auxin-related phenotypes of the *pi4kß1ß2* mutant. (**a**) primary root length of 11-day-old seedlings in response to different IAA concentrations, n = 22. Central line of the boxplots represents the median, circles represent individual values; *p*-value is indicated for significantly different groups; *t*-test with correction for multiple comparisons; (**b**) Elongation rate of primary root under gravistimulation, n = 10; (**c**) root tip orientation angle, n = 10; (**b**,**c**) gravitropic assay, 5-day-old seedlings were rotated to 90° on a horizontal microscope, images were taken every hour. Asterisks indicate statistically significant differences between genotypes, p < 0.05, paired *t*-test with correction for unequal variances. Experiments were repeated three times; data from a representative repeat are shown.

Mutant and WT 5-day-old seedlings were transferred to a cultivation medium containing various phytohormones. Seven days later, the lengths of the primary root, of the meristem and of the cortical cells within the differentiation zone were measured. The presence of IAA led to a decrease in the root length of WT plants; the decrease was more than 60% at 100 nM IAA. The *pi4kß1ß2* mutant was less sensitive to the auxin treatment, the decrease being only 20% at the concentrations tested (Fig. 2a, Supplementary Fig. S3). A lower sensitivity to exogenous auxin was also detected at the cellular and/or tissue levels. At 50 nM IAA, the length of WT cortical cells showed a 30% decrease compared to the control, while the mutant was insensitive. At 1 µM IAA, the decrease in length of WT cortical cells was 50%, compared to the control, while the mutant remained insensitive (Supplementary Fig. S4a). Concerning meristem size, 100 nM IAA caused a 20% shortening of its length in WT seedlings but no response was observed for the *pi4kß1ß2* mutant; this difference in IAA sensitivity was still apparent even at 1 µM (Supplementary Fig. S4b). Interestingly, the sensitivity of primary root length to a cytokinin (BAP) or to salicylic acid (SA) did not differ between *pi4kß1ß2* and WT seedlings (Supplementary Fig. S4c,d), thus indicating a specific response to auxins.

We then focused on another auxin-related process, the response to gravistimulation. Interestingly, both root elongation (i.e. the distance that the root tip grew since the 0’ time-point) and root orientation (i.e. the angle between the root tip at current and 0’ time-point) were affected in the double mutant in due course of 12 h experiment (Fig. 2b,c Supplementary Fig. S5, Supplementary movie SM1 for WT, Supplementary movie SM2 for *pi4kß1ß2*).

### The transcriptome of *pi4kß1ß2* roots shows partial similarities to IAA-treated WT roots

In order to better detail the *pi4kß1ß2* root phenotypes, an RNAseq transcriptomic analysis of roots was performed (Supplementary table S2). It was found that 2517 and 3418 genes were either up- or down-regulated, respectively, in *pi4kß1ß2* roots compared to WT roots. To be more stringent, we then only considered the genes passing a threshold of log2-fold change of 1.5. On these genes we performed a Gene Ontology classification (Supplementary Fig. S6). Among the genes induced in *pi4kß1ß2* roots compared to WT, we found enrichment in genes encoding extracellular, plasma membrane, or cell wall localized proteins, and underrepresentation of genes encoding cytoskeleton or mitochondria-associated proteins. Interestingly, among the repressed genes, the cell wall-associated proteins were also enriched, while cytoskeleton-localized proteins were overrepresented. As for biological processes, we found enrichment in the categories of “response to stress”, “signal transduction”and “development” for both groups of genes. Results of the RNAseq analysis were confirmed by qPCR on a selection of genes (Supplementary Fig. S7). Among the genes most induced in *pi4kß1ß2* roots, we found several that were involved in response to hypoxia, oxidative stress and induced systemic resistance (Supplementary Fig. S8). A focus on genes involved in auxin transport or metabolism is displayed (Supplementary table S4). *GH3.12* (AT5G13320) and *GH3.3* (AT2G23170) are markedly up-regulated in *pi4kß1ß2* roots compared to WT ones.

**Figure 3:**
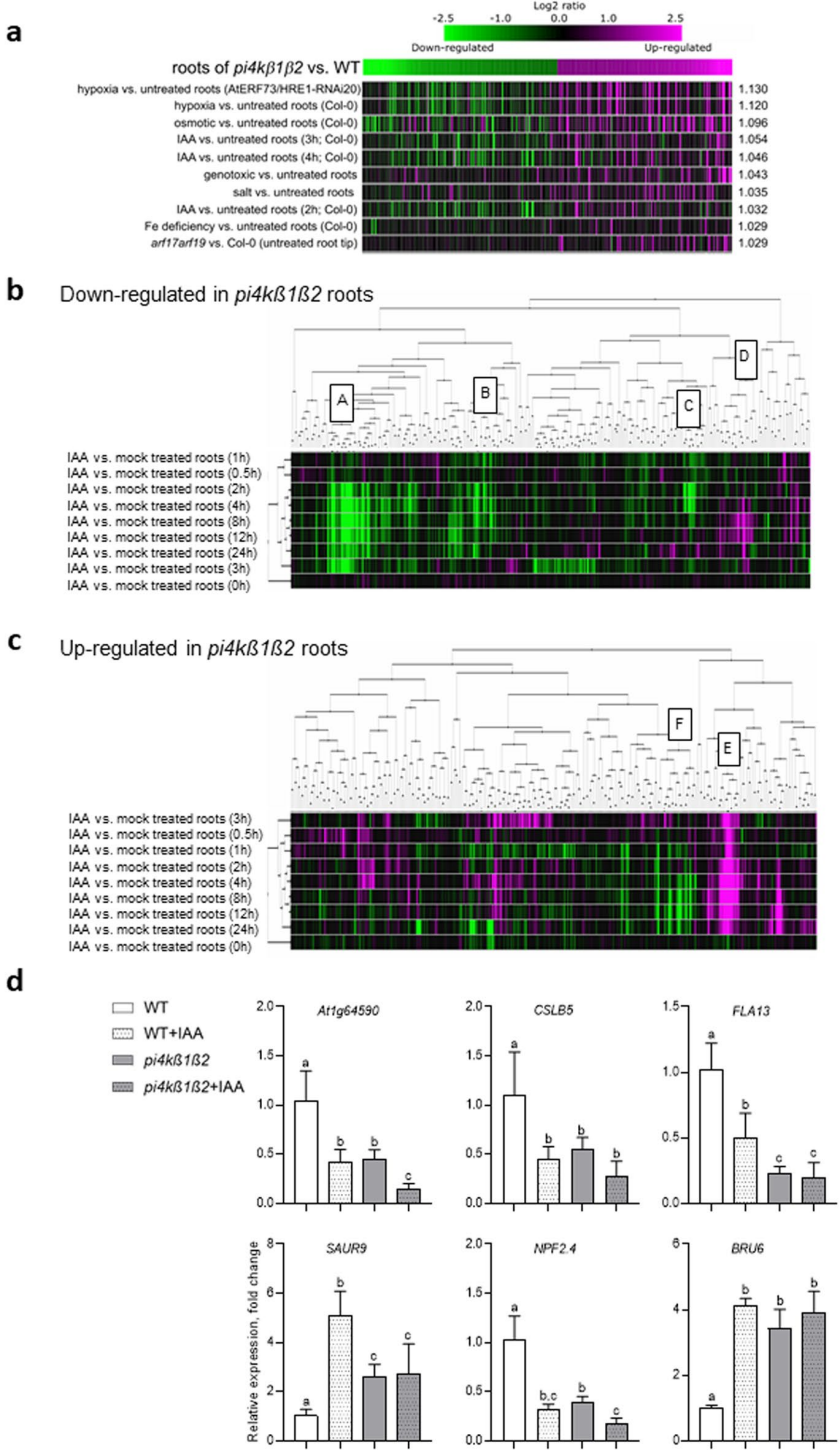
Transcriptomic analysis of *pi4kß1ß2* roots. (**a**) Similarity between the *pi4kß1ß2* roots transcriptome (compared to WT) and the stress-, hormone- or temperature- responsive transcriptomes. The 200 genes most up-regulated in *pi4kß1ß2* roots compared to the WT and the 200 genes most down-regulated in *pi4kß1ß2* roots compared to the WT were used as a signature to search for transcriptome experiments with the highest similarity. The similarity search was performed against the 56 root experiments classified as “stress”, “temperature” or “hormone” by Genevestigator (Hruz et al., 2008). Experiments were sorted according to Euclidean distance. Expression of the signature genes in the 10 most similar experiments are shown in color-scale; (**b,c**) Hierarchical clustering of curated root experiments dealing with the response to auxins. The 9 curated root experiments dealing with auxins in Genevestigator were retrieved. According to the expression in these experiments of the 200 most down-regulated (**b**) genes in our *pi4kß1ß2 vs. WT* root comparison, the genes and experiments were clustered with the Biclustering tool in Genvestigator. The same was done using the 200 most up-regulated (**c**) genes in our *pi4kß1ß2 vs. WT* root comparison. Similarities between expression profiles were determined using Pearson correlation. For each experiment, the duration of hormone treatment is indicated. Separated gene clusters with highest levels of induction/repression are labeled and genes are specified on the right panel; (**d**) Response of selected genes to auxin. Five-day-old seedlings were transferred to a medium containing 10 nM IAA, and roots for RNA extraction were harvested after 24 h. The data are presented in means ± SE, n = 9, with a Tukey honestly significant difference (HSD) multiple mean comparison post hoc test. Different letters indicate a significant difference (one-way ANOVA, Tukey HSD, *p*-value < 0.05).

Next, the list of the 200 most up-regulated and 200 most down-regulated genes in *pi4kß1ß2* mutant roots versus WT roots was used as a signature to interrogate public transcriptomic data using the Genevestigator similarity search program^25^. This was performed against curated root experiments dealing with root samples and classified as “Hormone”, “Temperature” or “Stress”. Out of the 10 most similar experiments, 7 concerned treatments with auxin (Fig. 3a). Within this set of curated root experiments (Fig. 3a), we then only selected the experiments dealing with response to auxins. According to the responses in these experiments of the 200 most repressed genes in our *pi4kß1ß2* versus WT root comparison, the experiments and the genes were clustered (Fig. 3b). This allowed the identification of clusters of genes, down-regulated in *pi4kß1ß2* mutant roots compared to WT ones and down-regulated in some experiments dealing with the response to auxin (Fig. 3b, clusters A,B,C; list of genes of these clusters in supplementary table 3). We did the same with the 200 most up-regulated genes in the *pi4kß1ß2* double mutant compared to WT roots and thus identified genes upregulated both in *pi4kß1ß2* mutant roots versus WT and up-regulated in curated experiments dealing with response to auxin in roots (Fig. 3c, cluster E). These clusters represent genes for which the effect of the *pi4kß1ß2* double mutation in the root compared to WT is similar to a treatment with auxin. Yet other clusters exist, consisting of genes that are down-regulated in *pi4kß1ß2* roots, but were shown to be upregulated by auxins in public transcriptomics data (Fig. 3b, cluster D; supplementary table S3), or genes that are up-regulated in *pi4kß1ß2* roots, but were shown to be upregulated by auxins in public transcriptomics data (Fig. 3c, cluster F; supplementary table S3). The transcript levels of selected auxin responsive genes representing different clusters were monitored by qPCR in mutant and WT plants, treated or not with 10 nM IAA for 24 h (Fig. 3d). The transcript level of *AT1G64590, CSLB5, SAUR9, NPF2.4* and *BRU6* in the untreated roots of *pi4kß1ß2* mutant was similar to that in WT roots treated with auxins. On the other hand, the transcription of *CSLB5, FLA13* and *BRU6* did not change in response to auxin in the *pi4kß1ß2* mutant, showing another evidence of affected auxin response.

### Assessing auxin sensitivity of *pi4kß1ß2* roots

**Figure 4:**
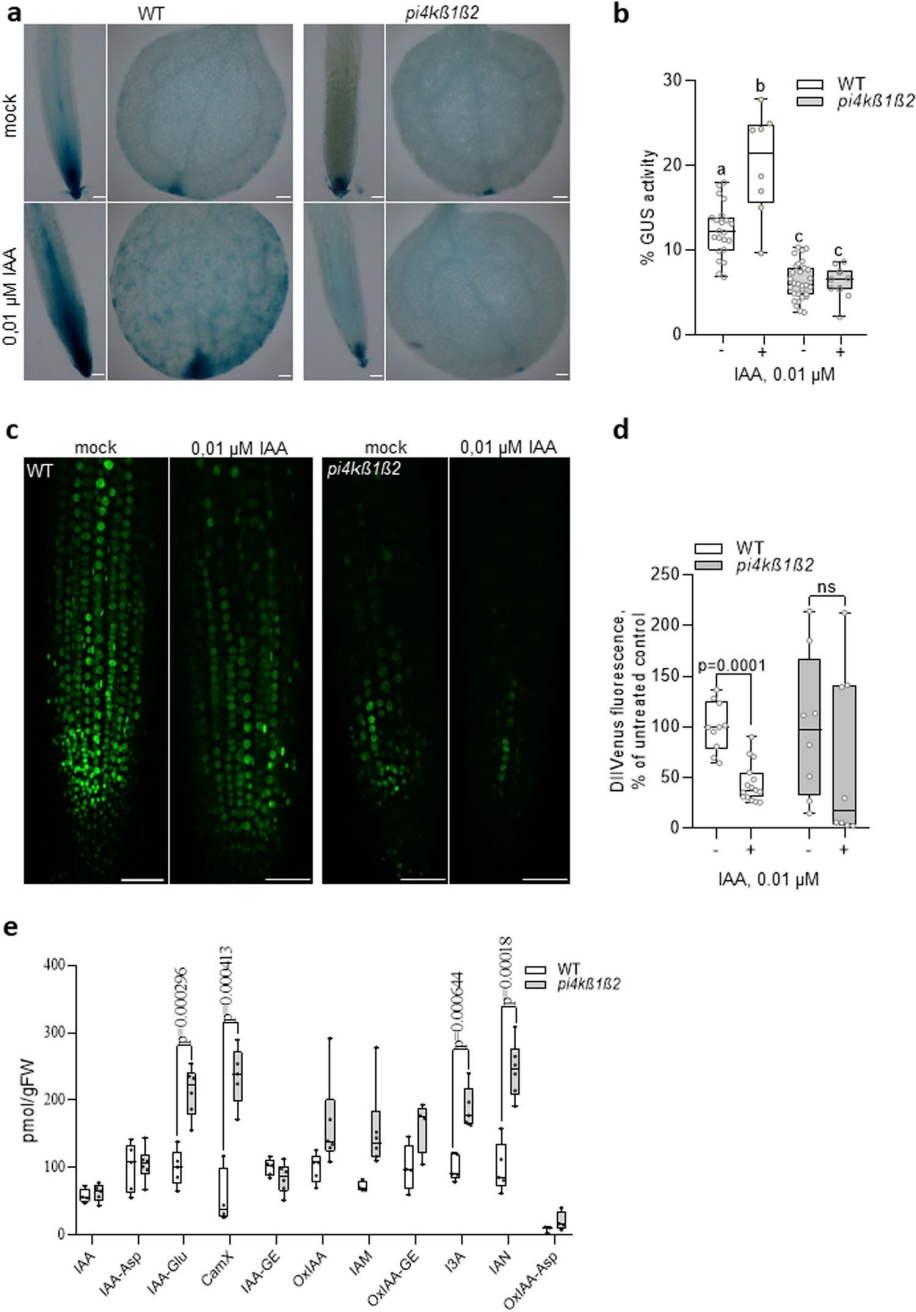
Auxin sensitivity of the *pi4kß1ß2* mutant. (**a**) representative images of DR5-GUS activity in 5-day-old root and cotyledons in the presence or not of 0.01 µM IAA for 12 h, scale bar = 100 µm; (**b**) DR5-GUS quantification, % of GUS-stained area in root meristem, n = 10; (**c**) representative images of DII-VENUS fluorescence in root tip of 7-day-old seedlings in the presence or not of 0.01 µM IAA for 1 h, maximum intensity Z-projections of 10 nm stacks, scale bar = 50 µm; (**d**) DII-VENUS fluorescence quantification, % of meristematic zone; n = 10; (**e**) quantitation of IAA metabolites and conjugates in 7-day-old roots, n = 6; Central line of the boxplots represents the median, circles represent individual values; *p*-value is indicated for significantly different groups, ns – non significant; unpaired *t*-test (**d,e**); The data are presented in means ± SD, n = 10, with a Tukey honestly significant difference (HSD) multiple mean comparison post hoc test. Different letters indicate a significant difference (one-way ANOVA, Tukey HSD, P < 0.05) (**b**); Experiments were repeated three times; data from a representative repeat are shown. IAA = indole-3-acetic acid, IAA-Asp = IAA-aspartate, IAA-Glu = IAA-glutamate, CamX = camalexin, IAA-GE = IAA-glucose ester, OxIAA = oxo-IAA, IAM = Indole-3-acetamide (IAA precursor), OxIAA-GE = oxo-IAA-glucose ester, I3A = indole-3-aldehyde, IAN = Indole-3-acetonitrile (IAA precursor), OxIAA-Asp = oxo-IAA-aspartate.

We next checked auxin transcriptional response by a reporter system, introducing by crossing the auxin sensitive synthetic promoter DR5^27^ fused to a GUS reporter gene into *pi4kß1ß2* background. Surprisingly, the basal level of DR5 promoter activity was lower in root and leaf meristem of the *pi4kß1ß2* plants (Fig. 4a,b). After exposure to 10 nM IAA, an important increase of DR5-GUS signal was detected in WT meristems, but not in the *pi4kß1ß2* mutant (Fig. 4a,b), confirming that the sensitivity to IAA is impaired in the mutant line.

The DII-VENUS^28^ construct was introduced into the *pi4kß1ß2* mutant by floral-dip agrobacterium transformation. DII-VENUS is a fast maturing form of a yellow fluorescent protein fused in-frame to the Aux/IAA-interaction domain (termed domain II;) and it is rapidly degraded in response to auxin^28^. It is used as a reporter of auxin level. As the DII-VENUS reporter was introduced by agrobacterium transformation, the potential positional effect of the insert cannot be excluded, so the basal fluorescent signal cannot be compared between the lines but signals can be compared within one line. After exposure to 10 nM IAA, a significant decrease of DII-VENUS fluorescence signal was detected in WT plants, but not in the *pi4kß1ß2* mutant (Fig. 4c,d). To check whether the mutant insensitivity to IAA might be a consequence of an elevated IAA level in control conditions, we extracted hormones from the total root system and measured the content of IAA metabolites and conjugates. No difference in the measured free IAA content was detected between genotypes, while IAA-Glu, CamX, I3A and IAN concentrations were higher in the *pi4kß1ß2* mutant than in the wild type (Fig. 4e).

### Localization of auxin efflux transporter PIN2 is altered in the *pi4kß1ß2* mutant

**Figure 5:**
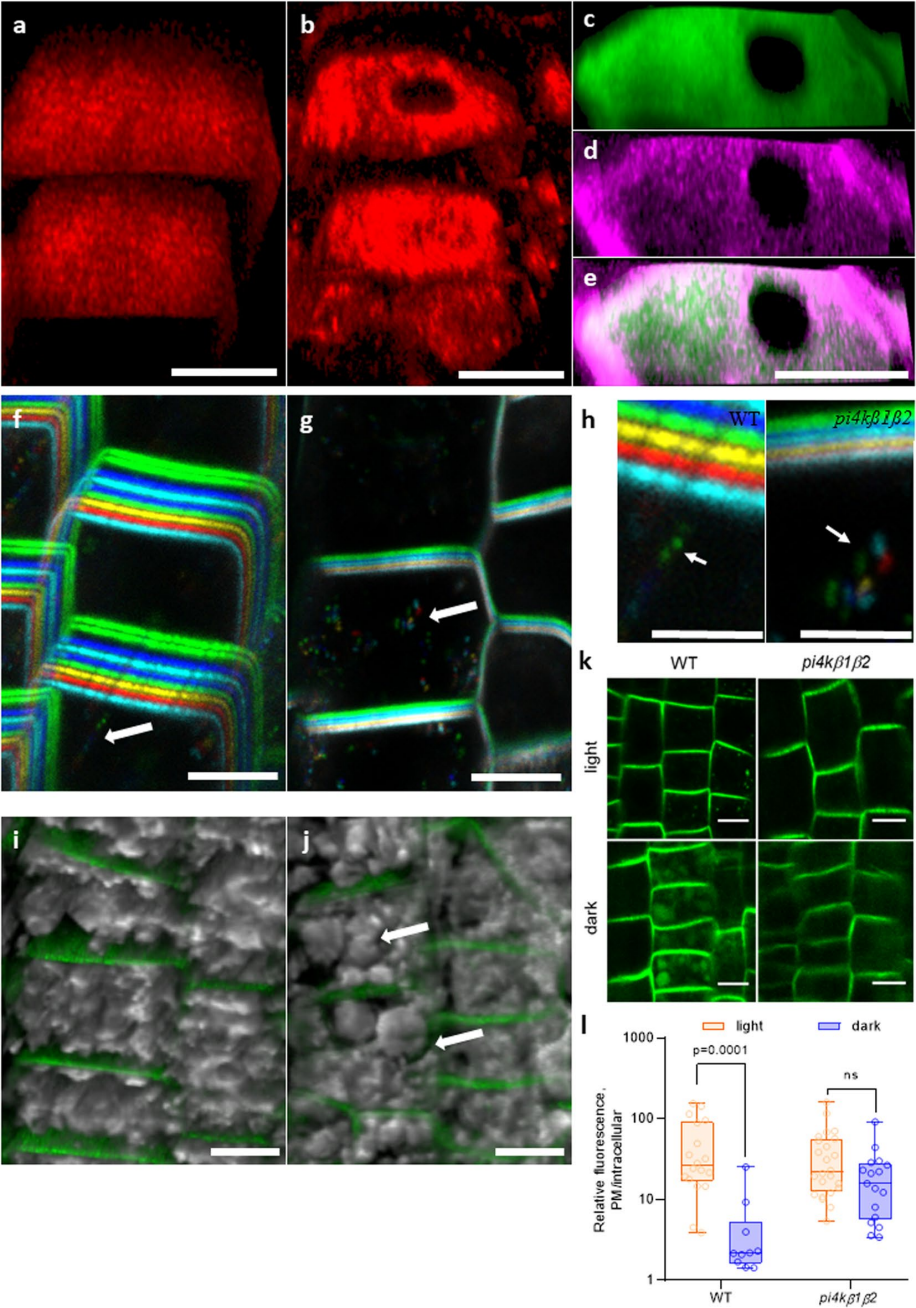
Visualization of PIN2-GFP subcellular distribution by confocal microscopy. (**a**) distribution of PIN2 along the PM in WT roots, immunostaining; (**b**) distribution of PIN2-GFP along the plasma membrane in *pi4kß1ß2* roots, immunostaining; (**c,d,e**) show PIN2-GFP signal overlapping with FM4-64 dye, (**c**, FM4-64; **d**, PIN2-GFP; **e**, merged signals); (**f,g**) color-coded projection of PIN2-GFP distribution and intracellular movement over time in (**f**) WT and (**g**) *pi4kß1ß2* backgrounds; arrows point to vesicles moving in time; (**h**), zoomed part of f and g, scale bars 5 μm, arrows point to vesicles moving in time; (**i,j**) merged 3D reconstruction of pictures taken along the z-axis of the bright field and fluorescent channel of PIN2 distribution along the plasma membrane and vacuole morphology in (**i**) WT and (**j**) *pi4kß1ß2* backgrounds; arrows point to enlarged vacuoles in *pi4kß1ß2*; (**k**) visualization of PIN2-GFP movement towards the lytic vacuole upon a dark shift of whole seedlings. After 1 h, the GFP signal was visible in the WT background, but not in *pi4kß1ß2*; (**l**) quantification of the GFP signal intensity in the lytic vacuole, each circle represents the plasma membrane/intracellular ratio for a single cell; *p*-value is indicated for significantly different groups, ns—non significant; unpaired *t*-test with correction for multiple comparisons; n = 25; scale bars 10 μm.

As auxin signaling is relying on the correct auxin transport between and within the cells, we investigated the localization and dynamics of auxin transporter PIN2. We analyzed plants expressing PIN2::PIN2-GFP by immunostaining (Fig. 5a–e) and confocal microscopy of PIN2-GFP in both WT and *pi4kß1ß2* backgrounds (Fig. 5f–k). Overall, PIN2-GFP was distributed on the plasma membrane in the same cell types and with a similar polar distribution in mutant roots compared to WT roots. However, in the *pi4kß1ß2* mutant, several “black holes” in the signal were detected along the plasma membrane (Fig. 5b–e, Supplementary Fig. S9). When counterstained with FM 4–64, a dye that labels the plasma membrane, it was seen that the unstained parts in the *pi4kß1ß2* roots corresponded to tunnels between adjacent cells (Fig. 5c–e). Confocal microscopy color-coded projections of pictures were taken over time to track PIN2-GFP intracellular movement in the meristematic zone. The chaotic distribution of vesicles in *pi4kß1ß2* compared to the vesicles aligned in WT showed not only differences in the amount of GFP-marked intracellular vesicles, but also that their movement was less rectilinear and very fast in *pi4kß1ß2* (compare Fig. 5f–h, where vesicles are indicated by white arrows, and the corresponding Supplementary movies SM3 for WT and SM4 for *pi4kß1ß2*). Differences in vacuolar morphology were also observed in *pi4kß1ß2* (Fig. 5i,j; Supplementary Fig. S10), with bigger and less fragmented vacuoles than the WT. When focused on growing root hair cells, altered movement of fluorescent marked vesicles in mature root hair cells and elongating root hairs in *pi4kß1ß2 PIN2::PIN2-GFP* was observed (Supplementary movies SM5 for WT and SM6 for *pi4kß1ß2*). Bright field imaging also revealed differences in the flow of cytoplasmic streaming. Circulation of the cytoplasmic stream occurred close to the plasma membrane and in a straight path in the WT, whereas in the mutant stream the stream flowed in less coordinated lanes. We then studied the response to a dark shift of whole seedlings, a treatment known to enhance PIN2-GFP delivery to the lytic vacuole^26^. A 1 h dark shift caused the translocation of PIN2-GFP to lytic vacuoles in WT roots but not in the double mutant (Fig. 5k,l). All these results point to altered intracellular trafficking dynamics in the roots of *pi4kß1ß2* seedlings.

### Actin stability and remodeling are affected in the *pi4kß1ß2* mutant

**Figure 6:**
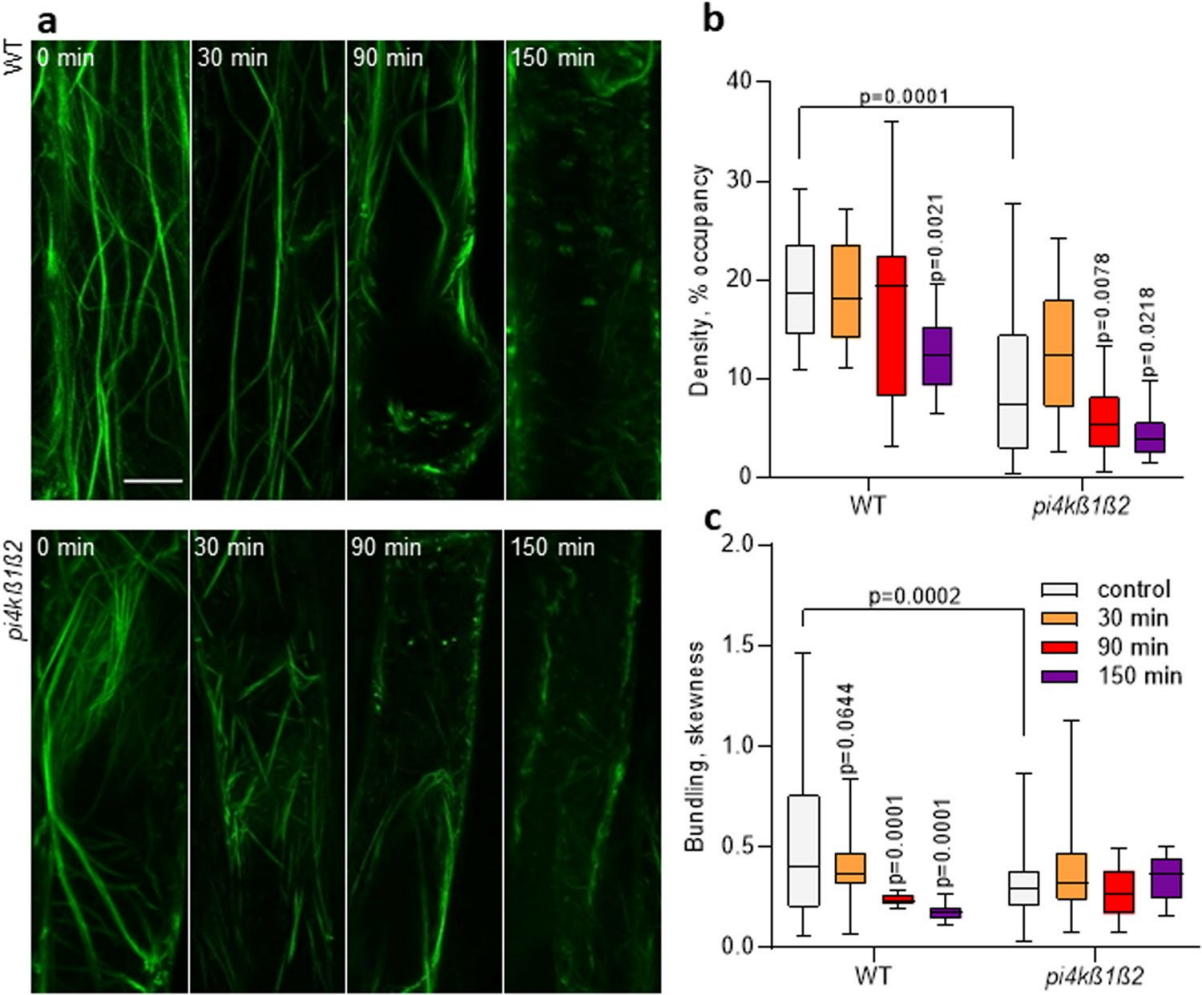
Actin reorganization in the *pi4kß1ß2* mutant in response to latrunculin B. Five-day-old seedlings expressing 35S::LifeAct-GFP were sprayed with 10 µM latB. (**a**) representative maximum intensity projections of root epidermis of WT and *pi4kß1ß2* plants; confocal microscopy, scale bar = 10 μm; (**b**) quantitative analysis of the density (expressed as percentage of occupancy) of actin filament arrays in epidermal cells; (**c**) quantitative analysis of the extent of filament bundling (expressed as skewness) in epidermal cells. Central line of the boxplots represents the median, plus represents the mean; circles represent individual values; *p*-value is indicated for significantly different time points within each genotype and for the comparison of genotypes immediately after treatment; one-way ANOVA with Tukey HSD *post-hoc* test; n = 10.

Five-day-old *pi4kß1ß2* seedlings expressing 35S::LifeAct-GFP were sprayed with 10 µM latB, a drug that inhibits actin polymerization. Treated seedlings were then observed under a confocal microscope (Fig. 6a). Without a latB treatment, the fluorescence signal occupancy was lower in *pi4kß1ß2* compared to WT seedlings. After a 90 min exposure to latB, the fluorescence signal occupancy in *pi4kß1ß2* decreased 40%, while no change was detected in WT plants (Fig. 6b). After a 150 min of exposure to latB, the signal occupancy in WT showed a 35% decrease compared to the control, while the occupancy decreased to 54% for the *pi4kß1ß2* mutant compared to control conditions. Interestingly, while WT roots showed a gradual decrease in actin filament bundling (Fig. 6c) in due course of latB treatment, no significant changes were observed in the *pi4kß1ß2* double mutant.

## Discussion

Here, we show that PI4Kß1ß2 deficiency led to up to a fourfold decrease of primary root length compared to WT seedlings. A dwarf phenotype, both in the roots and aerial parts, has already been reported for the *pi4kß1ß2* mutant^21^. Notably, the small rosette size of 4-week-old *pi4kß1ß2* mutant plants has been linked to an increased constitutive SA level^21^. Indeed, a *pi4kß1ß2sid2* triple mutant did not accumulate SA and it did not display the stunted rosette phenotype. However, *pi4kß1ß2sid2* seedlings still exhibited shorter roots than WT plants, thus showing that this root phenotype was a SA-independent process^21,22^. Furthermore, SA accumulation did not occur in young *pi4kß1ß2* seedlings^22^, thereby confirming that the root length phenotype was not due to high SA levels. Similar SA levels in *pi4kß1ß2* and WT roots were found in this work (Supplementary Fig. S11), thus confirming that the observed root phenotype was not related to altered SA levels and therefore it was an SA-independent process.

So what causes the short root phenotype of *pi4kß1ß2* seedlings? To answer this question, a detailed analysis of root morphology was undertaken (Fig. 1). The shorter primary roots of the double mutant appeared to be due to a reduced meristematic zone due to a lower number of cells. The CycB1::GUS associated signal occupied a significantly smaller (about 10%) area of the meristematic zone in *pi4kß1ß2* seedling roots when compared to the WT. This might explain in part why there were fewer cells in the meristematic zone of the mutant. An absent or a very short transition zone might also result from elevated auxin levels or an enhanced response to auxin. Indeed, the transition zone in a root begins where auxin levels attain a minimum^28^. The shorter primary root length in the *pi4kß1ß2* double mutant was also associated with smaller cortical cells measured in the differentiation zone. A reduced meristematic zone in the *pi4kß1ß2* mutant has been documented previously^29^, but we have supplemented these data by measuring meristem cell number and cell length in the differentiation zone. Concerning root hair length, we observed a bimodal distribution in the *pi4kß1ß2* plants, with very short hairs that gave the impression of bald zones. Interestingly, the regularity of trichoblasts/atrichoblasts formation was not affected in the mutant.

Due to the observed root phenotypes, an obvious next step was to assess the sensitivity of the double mutant to different hormones known to alter root growth. Root sensitivity to BAP or SA did not differ between *pi4kß1ß2* and WT seedlings. On the contrary, a loss of sensitivity in the double mutant to exogenous IAA was observed with respect to inhibition of primary root length, inhibition of cortical cell elongation, and elongation of the meristematic zone (Fig. 2, Supplementary Fig. S4a,b). This was in agreement with the experiments of Löfke et al., 2015, showing that altered vesicular trafficking due to inhibited PI4Kß1ß2 activity resulted in lower sensitivity to auxin NAA, altered vacuolar morphology and cell elongation^30^. Interestingly, *pi4kß1ß2* double mutant was less efficient in response to gravistimulation, another auxin-related process. Notably, not only the root elongation, but also the root tip orientation towards gravity vector were impaired in the mutant, suggesting gravity sensing defects.

To detail the cellular processes that were interfering with the response to stimulation in mutant roots, we performed full transcriptomic analysis. We found that differences in gene expression between *pi4kß1ß2* and WT roots were in part similar to those observed between auxin treated and non-treated WT roots. Yet, not all genes followed this trend and thus this similarity is only apparent. Besides, we tested by qPCR the response to auxin on a selection of genes, previously described as auxin responsive. The addition of exogenous IAA had no or only a small effect on the expression of those genes in *pi4kß1ß2* compared to the WT. Similarly, DR5-GUS was not induced in the *pi4kß1ß2* mutant after exposure to exogenous IAA. Besides, we also monitored a lower activity of the transcriptional reporter DR5-GUS in non-treated *pi4kß1ß2* mutant meristems (in root tip or cotyledon tip) compared to wild type ones. Therefore, there seems to be a lower sensitivity to auxin, either exogenous or endogenous, as far as gene expression is concerned.

Why is the double mutant less sensitive to exogenous IAA? A possibility could be that the mutant is no longer responsive because of a constitutive high auxin level. As mentioned above, some of the observed root traits of the double mutant (such as a very short transition zone) already resemble an auxin response even in non-treated conditions. Yet, the DR5-GUS in control conditions does not plead for higher IAA content in the mutant. Besides, the level of free bioactive IAA was comparable between mutant and WT roots. The use of the DII-VENUS, a reporter directly related to the bioactive signal itself^31^, gives another block of valuable information. DII corresponds to the auxin binding domain of AUX/IAA ; when IAA binds to this domain, AUX/IAA proteins are released from ARF factors and they can interact with SCF^TIR1^ that ubiquitinylates them for degradation by the proteasome. Because the reporter was introduced by transformation in the *pi4kß1ß2* mutant, we cannot directly compare data obtained in wild type to mutant background, but we can compare data within one line. In the mutant background, the DII-VENUS fluorescence was not significantly reduced by addition of exogenous IAA, as it was in WT seedlings. This is coherent with a non- or low- sensitivity to auxin we already documented based on gene expression data. The point is therefore to understand why DII-VENUS fluorescence is not reduced by addition of exogenous IAA. An explanation might be related to auxin homeostasis. In the *pi4kß1ß2* mutant, we detected elevated levels of several inactive auxin metabolites including the glutamate-conjugate form. IAA-Glu is an early metabolite and storage form of IAA and is synthesized upon incubation of plants with high concentrations of IAA, and is considered precursors for auxin degradation^32^. High level of IAA-Glu might result from a constitutive conjugation activity in the double mutant. Transcripts of different GH3 enzymes involved in aminoacid conjugation are indeed markedly up-regulated in the *pi4kß1ß2* roots. The affected balance between precursors or conjugates or IAA might then explain partial auxin-like response in roots. Conversely, this affected balance also probably reflects the affected sensitivity to active auxin. It is likely that in *pi4kß1ß2* mutant, exogenous IAA is conjugated, resulting in the IAA level not changing, as seen by DII-Venus monitoring.

The *pi4kß1ß2* mutant also showed an altered subcellular trafficking behaviour of PIN2, including trapping of the PIN2-GFP fusion protein in rapidly moving vesicles and a reduced transport towards the lytic vacuole upon a dark shift of *pi4kß1ß2* seedlings. Differences in *pi4kß1ß2* vacuolar morphology were also observed, with bigger and less fragmented vacuoles compared to the WT. This phenotype corresponds to that observed when WT Arabidopsis were treated with wortmannin, an inhibitor of PI4K activity^33^. In *pi4kß1ß2* roots, PIN2 localization by immunostaining and staining with FM64 evidenced “black holes” or stubs corresponding to tunnels between adjacent cells, also referred to as “cell wall stubs”. This can be linked with unfinished cytokinesis^23,34^. Caillaud et al. (2008)^35^ demonstrated that map65-3/ple mutants displayed cell wall stubs and multiple nuclei in the root meristem, both features of cytokinesis-defective mutants. Interestingly, MAP65-3 is a downstream target for inhibition by MAP kinase MPK4, and also a physical interaction between PI4Kβ1 and MPK4 has been reported^23^. Lin et al., (2019)^23^ proposed that PI4Kβ1 and MPK4 influence localization and activity of MAP65-3, acting synergistically to control phragmoplast dynamics. The altered cytoskeleton organization in our mutant could explain some of the trafficking issues, as the movement of membrane vesicles depends on the cytoskeleton^36^. For example, it was shown that PIN1 cycling is actin-dependent^37^. Proper assembly of the cytoskeleton in concert with the molecular motors, myosins, is essential for active internal transport, and therefore proper distribution of cargos, like PIN2^1,38^. Rho proteins mediate signals for cytoskeletal reorganization and cell polarity and they are also implicated in the regulation of endo- and exocytosis, and correct localization of PIN1^39^. Moreover, a direct interaction between both PI4Kβ1 and PI4Kβ2 and another GTPase protein involved in membrane trafficking, RabA4B, has been reported^40^. Membrane recruitment of ROP-GTPase ROP6 (and possibly also other ROPs) is also dependent on anionic phospholipids^41,42^. We therefore suggest that an altered regulation of Rho proteins by phosphoinositides could help explain, in part, the observed problems of *pi4kß1ß2* roots.

Can the alteration in trafficking and cytoskeleton explain the insensitivity to auxin? Proper spatial and temporal distribution of auxin in the root tip ensures differentiation of cell files and therefore conditions normal root hair outgrowth. This relies on an auxin efflux carrier modulating auxin circulation within root tip^1^. Therefore, altered trafficking can have consequences on root response to endogenous auxin. Concerning exogenously added auxin, the temporal distribution might not be the key factor and as mentioned above, auxin homeostasis probably explain the phenotypes.

Similar root phenotypes have been observed already in mutants affected in other steps of phosphoinositide turn-over. The *pip5k1pi5k2* mutant lacking two isoforms of PI4,5 K also showed shorter roots, a reduced meristematic zone and a lower sensitivity to exogenous auxins^43^. The *pip5k2* mutant has less lateral root formation and impaired gravitropism^43,44^. On the contrary, our *pi4kß1ß2* mutant has more lateral roots^45^. We can thus speculate that some of the observed phenotypes of *pi4kß1ß2* seedlings are in part due to altered production of PI4,5P2. As mentioned earlier, *pi4kß1ß2* roots did not respond to a gravitropic stimulus, which is dependent on fine-tuned spatial and temporal modulation of PIN2 distribution as well as auxin gradient regulation^46–49^. On the other hand, root bending requires a functional cytoskeleton network^49^. Proper actin cytoskeleton assembly is also required to trigger and maintain root hair integrity^50,51^, which is compromised in the *pi4kß1ß2* mutant. Furthermore, phosphoinositides can regulate actin dynamics by direct interaction with actin-binding proteins (ABPs)^52,53^, or affect actin polymerization, dynamics, and association with membranes indirectly through regulation of the activity and localization of Rho GTPases^54^ or via recruiting scaffolding proteins to the PM^52,55^. Indeed an altered actin cytoskeleton was observed in our mutant and phosphoinositides are well known to regulate actin organization^56^. A *pi4p5k10/11* double mutant also displayed an increased sensitivity to latB, whereas PI4P5K10 overexpression resulted in aggregation of the apical actin fringe in tobacco pollen tubes^43^.

Nevertheless, the intermediate signaling links connecting PI4Kbeta 1/2 deficiency and the resulting misregulation of PI4P on endomembranes with altered ectopic auxin signaling activities remain to be clarified. For instance, root gravitropic growth requires establishing of PIN2 polarity, that also involves MEMBRANE ASSOCIATED KINASE REGULATOR 4, MAKR4, acting downstream of auxin receptors TRANSMEMBRANE KINASE1 (TMK1). At the same time, PIN2 and MAKR4 plasma membrane localisation is dependent on anionic phospholipids turnover^57^*.* On the other hand, root bending requires cell wall modification that is under control of AHA H^+^-ATPases in cooperation with TMKs^58^. Interestingly, a mutant *cngc2*, deficient in plasma membrane-localized CYCLIC NUCLEOTIDE-GATED ION CHANNEL 2 exhibits a phenotype, partially similar to *pi4kß1ß2.* This concerns stunted root and rosette growth, SA accumulation in the leaves, lower gravitropic bending and impaired sensitivity to exogenous auxin*,* but, unlike *pi4kß1ß2, cngc2* mutant accumulates higher endogenous IAA^59^.

**Figure 7:**
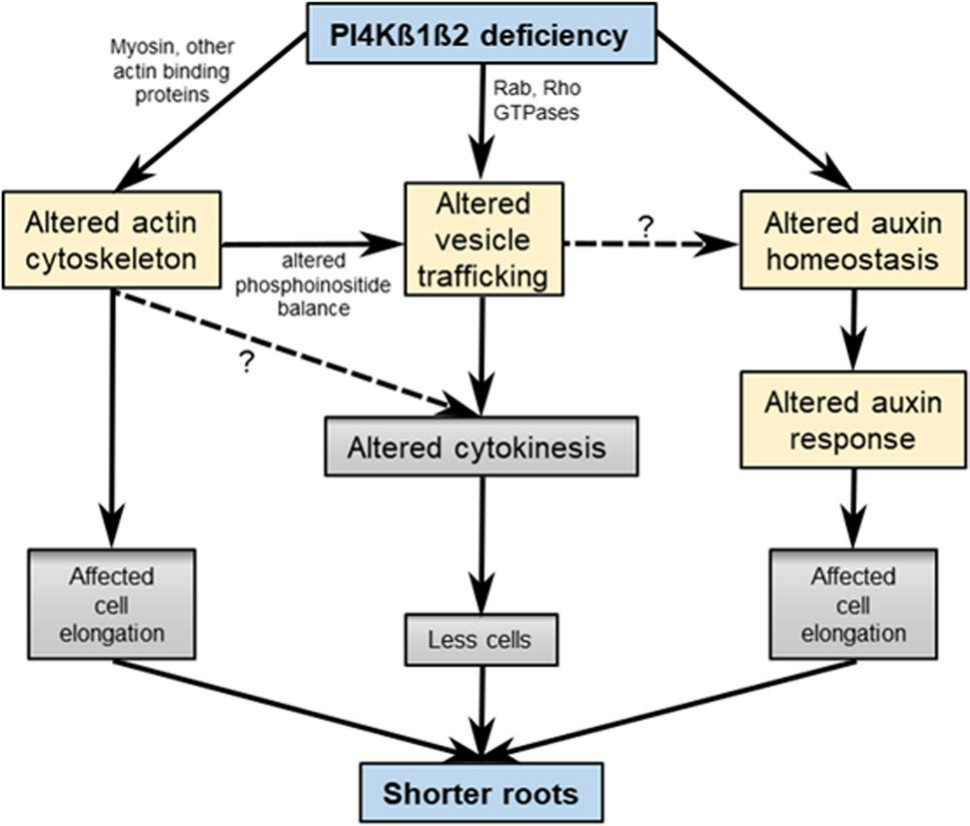
Working model for the impact of *pi4kß1ß2* mutations on root length. The *pi4kß1ß2* mutations lead to an altered actin cytoskeleton, an altered vesicle trafficking and an altered sensitivity to auxin including at the gene expression level. Altered trafficking can be linked to PI4K interacting with small G proteins like Rab or Rho proteins; it could also be a consequence of the weakened cytoskeleton. It is hypothesized that both altered cytoskeleton and trafficking prevent a correct cytokinesis. Finally, we propose that the short root phenotype results from multiple causes: altered actin cytoskeleton, altered cytokinesis, altered trafficking, and altered auxin responses.

Based on our observations, a working model is proposed that assembles multiple causes leading to the short root phenotype of the *pi4kß1ß2* mutant that arises from several root developmental defects, including reduced cell number and length (Fig. 7). Many correlate with altered dynamics of intracellular delivery processes. Plasma membrane establishment remains incomplete, cell architecture is misshaped, and PIN2 turnover is altered in the root elongation zone. This can be associated with a lower stability of the actin filaments network. Based on DII-VENUS degradation and gene expression, there appears to be a lack of response to auxin, endogenous or exogenous, in the *pi4kß1ß2* mutant. A link between altered trafficking/cytoskeleton integrity and this lack of gene expression response will require further investigations.

## Materials and methods

### Plant material

Experiments were performed using *A. thaliana* Col-0 as the WT control and the following mutant lines: *pi4kβ1β2* (SALK_040479/SALK_09069^30^), ^60^, CycB1::GUS^60^, DR5::GUS^27^, PIN2::PIN2-GFP^43^, PIN2::PIN2-GFP in a *pi4kβ1β2* background^23^, DII-VENUS^28^, DII-VENUS in *pi4kβ1β2* (this study), 35S::LifeAct-GFP, 35S::LifeAct-GFP in a *pi4kβ1β2* background^61^. The DII-VENUS construct was introduced into *pi4kβ1β2* by floral dip transformation; three independent lines were selected and the T4 generation was studied. CycB1::GUS^60^ and DR5::GUS^27^ constructs were introduced into the *pi4kβ1β2* background by crossing, and homozygous F3 seeds were used. Genotyping primers are listed in Supplementary Table 1. This study complies with relevant institutional, national, and international guidelines and legislation for using plant material. Analytical grade chemicals used in this study were purchased from Sigma Aldrich (St. Louis, Missouri, USA).

### Plant cultivation

Seeds were surface sterilized with 1.6% sodium hypochlorite (30% of SAVO®, Unilever) solution containing 0.02% (v/v) TWEEN20 (Sigma Aldrich, St. Louis, Missouri, USA). Seeds were stratified for 2 days at 4 °C in the dark. Seeds were germinated for 3 days in Petri dishes containing half-strength Murashige–Skoog basal salt medium (Duchefa, Haarlem, Netherlands), pH 5.7, supplemented with 1% (w/v) sucrose and 0.8% (w/v) plant agar (Duchefa, Haarlem, Netherlands) at 22 °C under a 16 h light/8 h dark regime in a vertical position.

For the primary root length analysis, 4 days after germination, seedlings were transferred to square Petri plates containing the same medium supplemented or not with hormones (IAA at 0.05, 0.1 or 1 µM final concentration; BAP, at 0.1, 0.5, 1 or 5 µM; SA at 2, 10 or 20 µM). Stock solutions at 200 mM were prepared in distilled water and few drops of 1 N NaOH. After 7 days of cultivation in vertical position Petri dishes were scanned for the primary root length measurement (Epson Perfection V700 Photo, Suwa, Japan, at 600 dpi resolution). For the measurement of the lengths of meristem, elongation zone and cortical cells, roots were observed under an ApoTome Zeiss microscope with a 5 × objective at bright field settings. Images were analyzed with FiJi software^62^. At least 12 seedlings were analyzed for each variant. For the measurement of root hair length and density, 5-day-old seedlings were photographed under a stereo microscope (SteREO Discovery V8, Carl Zeiss GmbH, Jena, Germany) equipped with an AxioCam HRc camera. Images were imported into FiJi software and root hair length was measured manually using a segmented line tool. At least 60 root hairs from 10 seedlings were analyzed for each variant. For PIN2 localization and dynamics analysis, DII-Venus assay or actin structure evaluation, 7-days old seedlings were used. For actin structure evaluation, seedlings expressing 35S::LifeAct-GFP were sprayed with 10 μM latB (latrunculin B) for different time incubations (30 min, 90 min and 150 min) and were used for confocal microscopy. For DII-VENUS assay, seedlings were transferred to the media supplemented 0.01 μM IAA for 1 h and subjected to microscopy. The fluorescence intensity of nuclei was extracted using FiJi software.

### Gravitropic test

Gravitropic response test was performed as previously described^46^. Five-day-old seedlings were transferred onto fresh Petri Dishes containing half-strength Murashige–Skoog basal salt medium (Duchefa, Haarlem, Netherlands), pH 5.7, supplemented with 1% (w/v) sucrose and 0.8% (w/v) plant agar (Duchefa, Haarlem, Netherlands) and aligned in a horizontal orientation. Plants were scanned at indicated time points using a Horizontal LSM880 with Airyscan module for 12 h and images were used to determine root reorientation. The root turning angle and length were calculated for each time point. Ten roots were imaged for each genotype.

### GUS staining

GUS staining was performed as previously described^63^. Briefly, 4- or 8-day-old seedlings were incubated in 2 mM X-Gluc, 50 mM NaH_2_PO_4_, pH 7, 0.5% (v/v) Triton-X, 0.5 mM K-ferricyanide, for 16 h at 37 °C. Chlorophyll was removed by repeated washing with 80% (v/v) ethanol. Imaging was performed using an ApoTome Zeiss microscope with a 5 × objective at bright field settings.

### Confocal microscopy

A Zeiss LSM 880 inverted confocal laser scanning microscope (Carl Zeiss AG, Germany) was used with a 40 × C-Apochromat objective (NA = 1.2 W). Fluorescence signals were processed with Zen Blue software (Zeiss), where PIN2 distribution was evaluated as a ratio of mean fluorescence intensity at the apical PM to mean intracellular fluorescence intensity of individual cells. Fluorescence associated with actin filaments (LifeAct-GFP) or DII-VENUS was acquired by excitation at 488 nm and emission at 490–540 nm for GFP. Images were acquired in z-stacks (step size 0.43 μm, 40–50 sections per stack). Actin filaments density and DII-VENUS signal intensity was calculated by FiJi software as the percent occupancy of GFP signal in each Maximum intensity projection. For each variant, fluorescent intensity of at least 5 roots was analyzed with 1–5 ROI (region of interest) per 1 root (ROI corresponds to one entire cell for actin; ROI corresponds to meristematic zone for DII-VENUS). For analyzing the skewness, all z-stack images were skeletonized and projected using a plugin moment calculator.

For tracking PIN2-GFP distribution in WT and *pi4kß1ß2* over time (supplementary movies SM3 and SM4), ten frames were continuously obtained by confocal microscopy to track the movement of PIN2:GFP in root epidermis cells in the transition zone and compiled to a movie. PIN2-GFP subcellular distribution and cell properties were monitored on a Zeiss LSM880 microscope (AxioObserver, objective C-Apochromat 40x/1.2 W Korr FCS M27, Filter 493–598, Laser 488 nM, using zoom factor 6. Original picture size is 35,42 μm × 35,42 μm, scale bar is 10 μm.

For root hair video showing cytoplasmic streaming (supplementary movies SM5 and SM6)*,* maximum intensity projections of a Z-stack of a root hair were taken over time. Fluorescent channel and bright field are presented together. Fluorescent channel: visualization of cytoplasmic streaming in root hair cell outgrowing a root hair, based on differential movement of fluorescent intracellular structures in the line PIN2::PIN2-GFP compared to the mutant expressing PIN2-GFP. The movie was reconstructed from confocal pictures captured in 20 frames (time-lapse) and in 18 (WT background)/19 slices (mutant background) through the root hair along the z-axis. Original picture size is 106.27 μm × 106.27 μm, pictures were captures with EC Plan-Neofluar 20x/0.50 (WD = 2.0 mm) objective, using zoom factor 4. Scale bar is 10 μm. Brightfield channel: visualization of cytoplasmic streaming in a movie reconstructed from confocal pictures captured in 20 frames (time-lapse) and in 18 (WT background)/19 slices (mutant background) along the root hair in the z-axis. Original picture size is 106.27 μm × 106.27 μm, pictures were captures with EC Plan-Neofluar 20x/0.50 (WD = 2.0 mm) objective, using zoom factor 4. Scale bar is 10 μm.

### FM 4–64 staining of the plasma membrane

Five-day-old *A. thaliana* seedlings expressing PIN2::PIN2-GFP^47^ were incubated with 2 μM FM 4–64 (Molecular Probes, catalogue number T13320) in half-strength Murashige and Skoog (Sigma Aldrich, St. Louis, Missouri, USA) liquid medium in multi-well plates for 5 min and then rinsed 3 times in liquid medium^64^. The seedlings were observed using a confocal scanning microscope Zeiss LSM 880 with Airyscan module.

### PIN2 immunolocalization

Whole mount immunolocalization of 5–day–old seedlings was performed as described previously^65^ with minor changes. The protocol was adapted to the InSituPro VS liquid-handling robot (Intavis AG, Germany). Prior to immunolocalization, seedlings were fixed 1 h with 4% paraformaldehyde dissolved in MTSB (50 mM PIPES, 5 mM EGTA, 5 mM MgSO_4_·7H_2_O pH 7, adjusted with KOH), at room temperature, with no vacuum. In the robot, the procedure started with several washes with MTSB-T (MTSB + 0.01% TritonX-100) then cell walls were digested with 0.05% Pectolyase Y-23 in MTSB-T and membranes were permeated with DMSO/Igepal in MTSB-T. Samples were blocked with BSA (blocking solution: 2% BSA in MTSB-T) and incubated first with anti-PIN2 rabbit antibody (kindly provided by Prof. C. Luschnig, dilution 1:500) and then a secondary anti-rabbit Alexa Fluor 546 antibody (Thermo Fisher Scientific, dilution 1:1000). Both antibodies were diluted in BSA. Between the described steps, washes with MTSB-T were provided and at the end MTSB-T was exchanged for deionized water. Seedlings were then transferred from the robot to 50% glycerol in deionized water and the fluorescence signal was measured using a confocal scanning microscope Zeiss LSM 880 with Airyscan module.

### RNA extraction and RNA-seq

For each of the 3 biological repetitions, RNA samples were obtained by pooling RNAs from more than 70 plants. Seven-days-old seedlings roots (100–200 mg fresh weight) were frozen in liquid N_2_. Roots were homogenized in tubes with 1 g of 1.3 mm silica beads using a FastPrep-24 instrument (MP Biomedicals, USA). Total RNA was isolated using a Spectrum Plant Total RNA kit (Sigma-Aldrich, USA) and treated with a DNA-free kit (Ambion, USA). The quantity of extracted RNA was measured using NanoDrop.

Sequencing was carried out using an Illumina NexSeq500 (IPS2 POPS platform). RNA-seq libraries were made using the TruSeq Stranded mRNA kit (Illumina®, California, USA). The RNA-seq samples were Single End (SE) sequenced, stranded with a sizing of 260 bp and a read length of 75 bases, lane repartition and barcoding gave approximately 45 million SE reads per sample.

Gene transcription measurement was conducted as described previously^61^. In general, 1 μg of RNA was converted into cDNA with M-MLV RNase H−Point Mutant reverse transcriptase (Promega Corp., USA) and an anchored oligo dT21 primer (Metabion, Germany). Gene expression was quantified by qRT-PCR using a LightCycler 480 SYBR Green I Master kit and LightCycler 480 (Roche, Switzerland). The PCR conditions were 95 °C for 10 min followed by 45 cycles of 95 °C for 10 s, 55 °C for 20 s, and 72 °C for 20 s. Melting curve analysis was then conducted. CT values of target genes were normalized to the housekeeping gene TIP41. A list of the analyzed genes and primers is available in Supplementary table 1.

### RNA-seq bioinformatic treatments and analyses

To facilitate comparisons, each sample followed the same steps from trimming to counts. RNA-Seq preprocessing included trimming library adapters and performing quality controls. The raw data (fastq) were trimmed using the Trimmomatic^66^ tool for a Phred Quality Score Qscore > 20, read length > 30 bases, and ribosome sequences were removed with the sortMeRNA tool^67^.The genomic mapper STAR (version 2.7. 3a^68^) was used to align reads against the *A. thaliana* genome (from TAIRv10), with options–outSAMprimaryFlag AllBestScore–outFilterMultimapScoreRange 0 to keep the bests results. Transcript abundance of each gene was calculated with STAR and counts only single reads for which reads map unambiguously one gene, thus removing multi-hits. According to these rules, around 97% of SE reads were associated with a gene, 1–2% of SE reads were unmapped and 1.22–1.66% of SE reads with multi-hits were removed. Differential analyses followed the procedure previously described^69^. Briefly, genes with less than 1 read after a counts-per-million (CPM) normalization in at least one half of the samples were discarded. Library size was normalized using the trimmed mean of M-value (TMM) method and count distribution was modeled with a negative binomial generalized linear model. Dispersion was estimated by the edgeR method^70^ in the statistical software ‘R’^71^ (Version 3.2.5 R Development Core Team (2005). Expression differences compared 2 samples using likelihood ratio tests and *p*-values were adjusted with the Benjamini–Hochberg procedure to control False Discovery Rate (FDR). A gene was declared differentially expressed if the adjusted *p*-value < 0.05.

Genes were classified using the Classification SuperViewer Tool developed by^72^ as described previously^20^. The classification source was set to Gene Ontology categories as defined by^73^. The frequency of each category was normalized to the whole Arabidopsis set. The mean and standard deviation for 100 boot-straps of our input set were calculated to provide some idea as to over- or under-representation reliability. Similarity analysis were performed using tools developed by Genevestigator^29^. The “Hierarchical clustering” tool works on the expression matrix defined by a microarray experiment selection and a gene selection. The “Biclustering” tool identifies groups of genes that are expressed above or under a set threshold ratio in a subset of conditions rather than in all conditions.

### Hormone measurements

Whole roots (50–100 mg) were harvested from 7-day-old vertical grown seedlings. At least 6 samples were analyzed for WT and *pi4kβ1β2*. Hormone analysis was performed with a LC/MS system consisting of UHPLC 1290 Infinity II (Agilent, Santa Clara, CA, USA) coupled to 6495 Triple Quadrupole Mass Spectrometer (Agilent, Santa Clara, CA, USA), operating in MRM mode, with quantification by the isotope dilution method. Detailed methodology was described previously^74^.

### Data deposition

Experimental steps, from growth conditions to bioinformatic analyses, have been deposited in the CATdb database^75^ as ProjectID NGS2020_14_pi4kb1b2 and further submitted to the international repository GEO^76^ as ProjetID = GSE179635.

### Statistical analysis

At least three biological repetitions were carried out for all experiments, and at least 10 seedlings were analyzed for each treatment. Student’s *t*-test with correction for multiple comparisons and one-way ANOVA with Tukey’s HSD post-hoc test were applied; the exact number of values and statistical procedures are stated in the figure legends.

## Supplementary Information

Supplementary Information 12.

Supplementary Information 13.

Supplementary Information 14.

Supplementary Information 15.

Supplementary Video 1.

Supplementary Video 2.

Supplementary Video 3.

Supplementary Video 4.

Supplementary Video 5.

Supplementary Video 6.

Supplementary Information 16.
