# Supplementary material for: An Arabidopsis mutant deficient in phosphatidylinositol-4-phosphate kinases ß1 and ß2 displays altered auxin-related responses in roots
Source: Sci Rep. 2022 Apr 28;12:6947. doi: 10.1038/s41598-022-10458-8 (PMC9051118; doi:10.1038/s41598-022-10458-8)

SUPPORTING INFORMATION

Supplementary table T1. Primers used in the study.

Supplementary table T2. List of genes differentially expressed in roots of *pi4kß1ß2* versus WT.

Supplementary table T3. Clusters of genes, down-regulated in pi4kß1ß2 mutant roots compared to WT ones and down-regulated in curated public transcriptomics experiments dealing with the response to auxin (clusters A,B,C); genes that are down-regulated in pi4kß1ß2 roots, but were shown to be upregulated by auxin in curated public transcriptomics experiments dealing with the response to auxin (cluster D); genes upregulated both in pi4kß1ß2 mutant roots versus WT and up-regulated in curated experiments dealing with response to auxin in roots (cluster E); genes upregulated in pi4kß1ß2 mutant roots versus WT but down-regulated in some curated experiments dealing with response to auxin in roots (F).

Supplementary table T4. List of auxin transporter and metabolism genes differentially expressed in roots of *pi4kß1ß2* versus WT.

Supplementary movie SM1. Response to gravistimulation in WT root. Fifty frames were continuously obtained by Graviscope Zeiss LSM800 (objective EC Plan-Neofluar 20x/0.50 M27, Filter 494-555, Laser 488 nM) to track the gravitropic response and compiled to a movie, scale bar is 200µm.

Supplementary movie SM2. Response to gravistimulation in *pi4kß1ß2* root. Fifty frames were continuously obtained by Graviscope Zeiss LSM800 (objective EC Plan-Neofluar 20x/0.50 M27, Filter 494-555, Laser 488 nM) to track the gravitropic response and compiled to a movie, scale bar is 200µm.

Supplementary movie SM3. Tracking PIN2:GFP distribution in WT over time. Ten frames were continuously obtained by confocal microscopy to track the movement of PIN2:GFP in root epidermis cells in the transition zone and compiled to a movie. PIN2:GFP subcellular distribution and cell properties were monitored on a Zeiss LSM880 microscope (AxioObserver, objective C-Apochromat 40x/1.2 W Korr FCS M27, Filter 493-598, Laser 488 nM, using zoom factor 6. Original picture size is 35,42µm x 35,42µm, scale bar is 10µm.

Supplementary movie SM4. Tracking PIN2:GFP distribution in *pi4kß1ß2* over time. Ten frames were continuously obtained by confocal microscopy to track the movement of PIN2:GFP in root epidermis cells in the transition zone and compiled to a movie. PIN2:GFP subcellular distribution and cell properties were monitored on a Zeiss LSM880 microscope (AxioObserver, objective C-Apochromat 40x/1.2 W Korr FCS M27, Filter 493-598, Laser 488 nM, using zoom factor 6. Original picture size is 35,42µm x 35,42µm, scale bar is 10µm.

Supplementary movie SM5. Root hair video showing cytoplasmic streaming in WT. Maximum intensity projection of a Z-stack of a root hair over time. Fluorescent channel and bright field together. Fluorescent channel: visualization of cytoplasmic streaming in root hair cell outgrowing a root hair, based on differential movement of fluorescent intracellular structures in the line PIN2::PIN2:GFP compared to the mutant expressing PIN2:GFP. The movie was reconstructed from confocal pictures captured in 20 frames (time-lapse) and in 18 (WT background)/19 slices (mutant background) through the root hair along the z-axis.

Original picture size is 106.27µm x 106.27µm, pictures were captures with EC Plan-Neofluar 20x/0.50 (WD=2.0mm) objective, using zoom factor 4. Scale bar is 10 µm.

Supplementary movie SM6. Root hair video showing cytoplasmic streaming in WT. Maximum intensity projection of a Z-stack of a root hair over time. Fluorescent channel and bright field together. Fluorescent channel: visualization of cytoplasmic streaming in root hair cell outgrowing a root hair, based on differential movement of fluorescent intracellular structures in the line PIN2::PIN2:GFP compared to the mutant expressing PIN2:GFP. The movie was reconstructed from confocal pictures captured in 20 frames (time-lapse) and in 18 (WT background)/19 slices (mutant background) through the root hair along the z-axis. Original picture size is 106.27µm x 106.27µm, pictures were captures with EC Plan-Neofluar 20x/0.50 (WD=2.0mm) objective, using zoom factor 4. Scale bar is 10 µm.

Supplementary fig. S1 Cortical cell length of 11-day-old seedlings of *A. thaliana* Col-0 (WT) and *pi4kß1ß2* mutant; Student t-test, n=200.

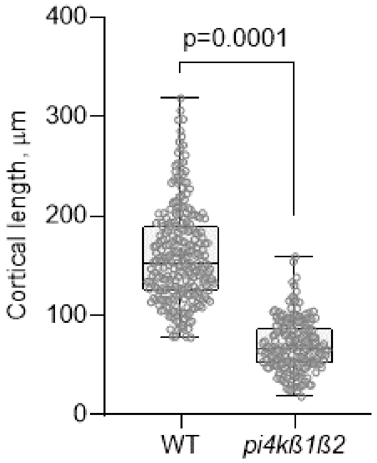

Supplementary fig. S2 Regularity of trichoblast (green) and atrichoblast (magenta) cell lines of 7-day-old seedlings of *A. thaliana* Col-0 (WT) and *pi4kß1ß2* mutant, scale bar 100 μm.

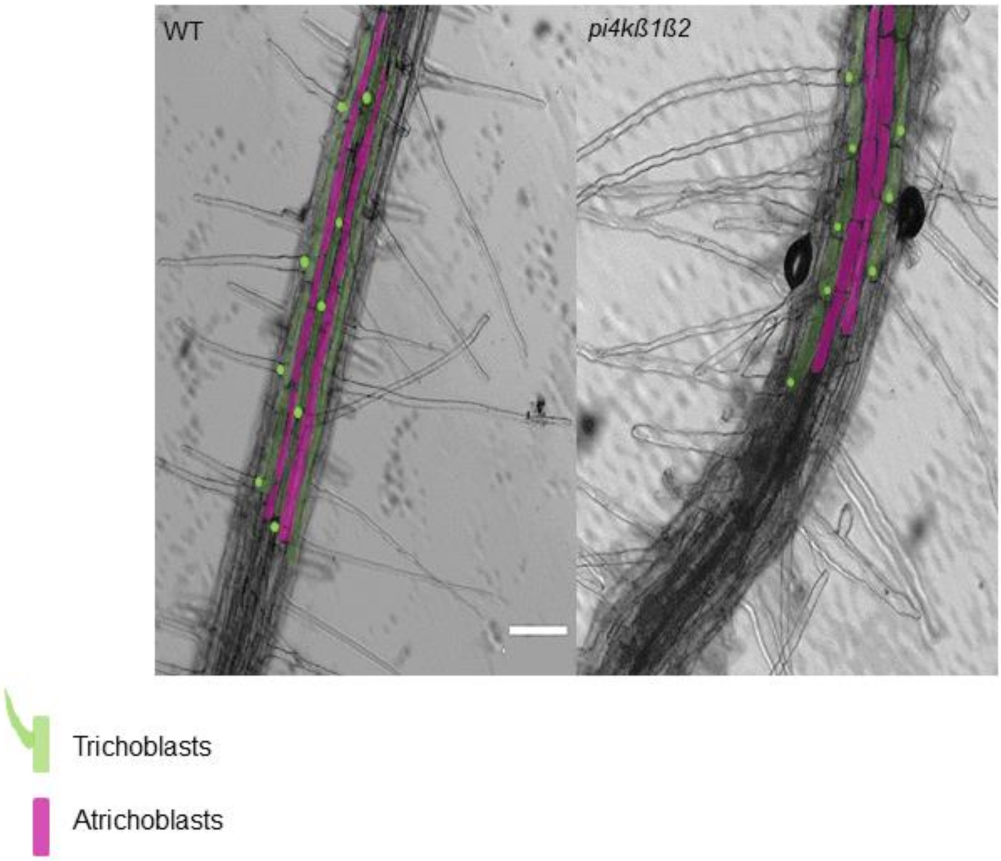

Supplementary fig. S3 Auxin-related phenotypes of the *pi4kß1ß2* mutant. Primary root length (absolute value) of 11-day-old seedlings in response to different IAA concentrations, n=22. P-value is indicated for variants significantly different from control with no IAA within each genotype, *t*-test with correction for multiple comparisons.

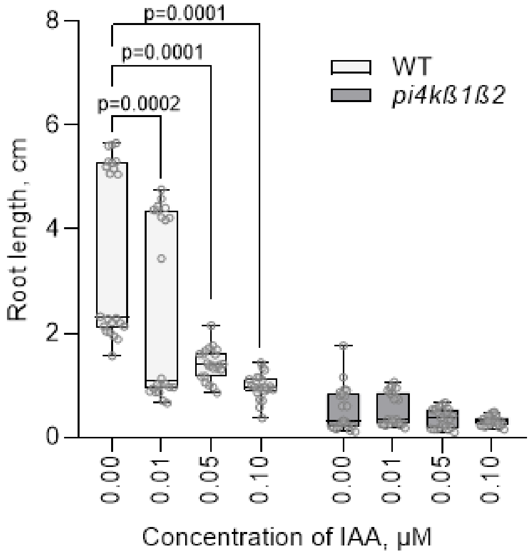

Supplementary fig. S4 Response to phytohormones of 11-day-old *A. thaliana* Col-0 (WT) and *pi4kß1ß2* mutant seedlings, 7 days after their transfer to square Petri plates containing the same medium supplemented or not with hormones. **a**, IAA, cortical cell length, n=22; **b**, IAA, meristem length, n=22; **c**, SA, primary root length, n=10; **d**, BAP, primary root length, n=10. Central line of the boxplot represents the median; circles represent individual values from three biological repeats. Different letters indicate variants significantly different in every growing condition; one-way ANOVA with Tukey-HSD post-hoc test.

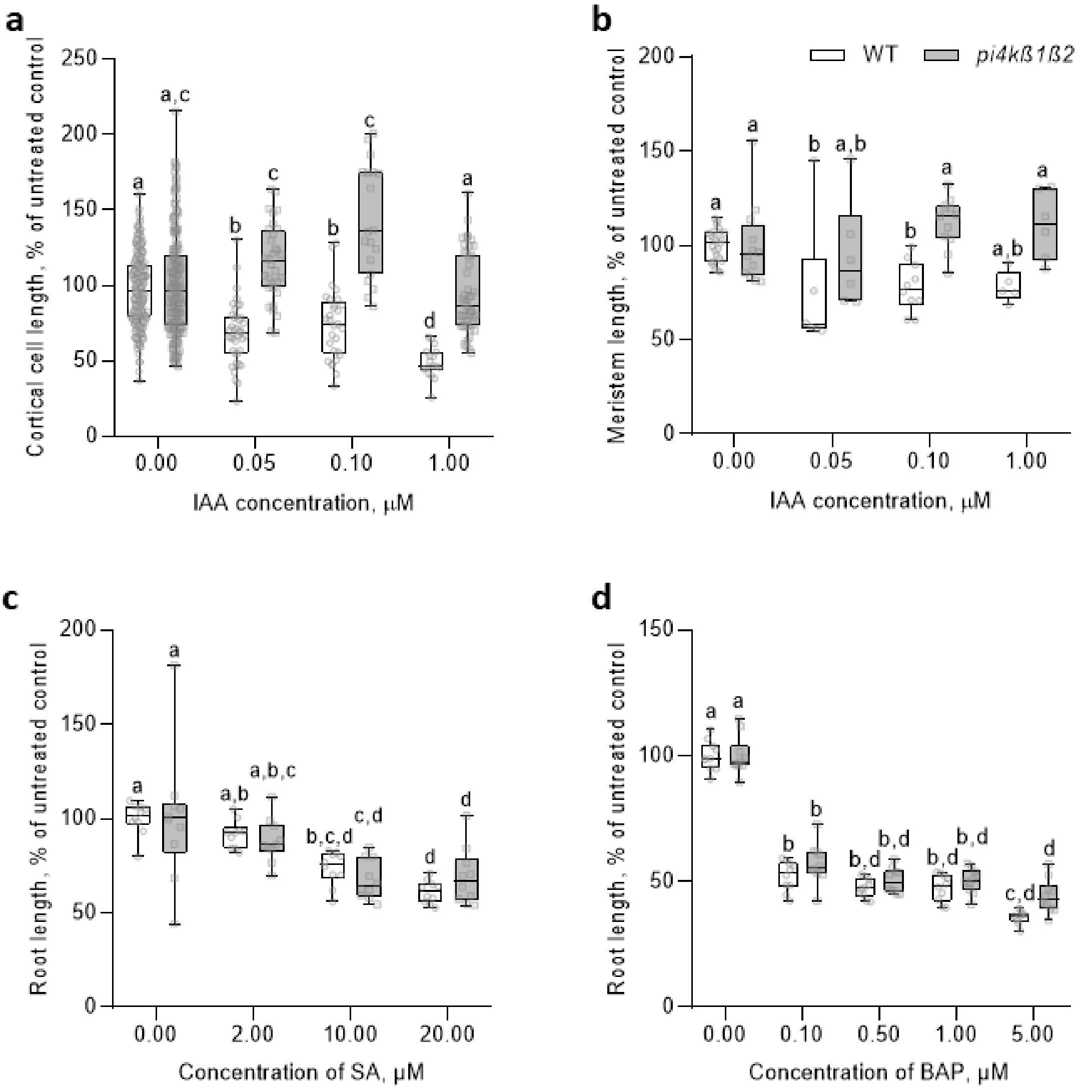

Supplementary fig. S5 Representative images of the gravitropic assay of 5-day-old seedlings of WT and *pi4kß1ß2* mutant. Seedlings cultivated vertically were rotated at 90° and imaged on a horizontal microscope for 7 h (one image per h), scale bar 200 µm.

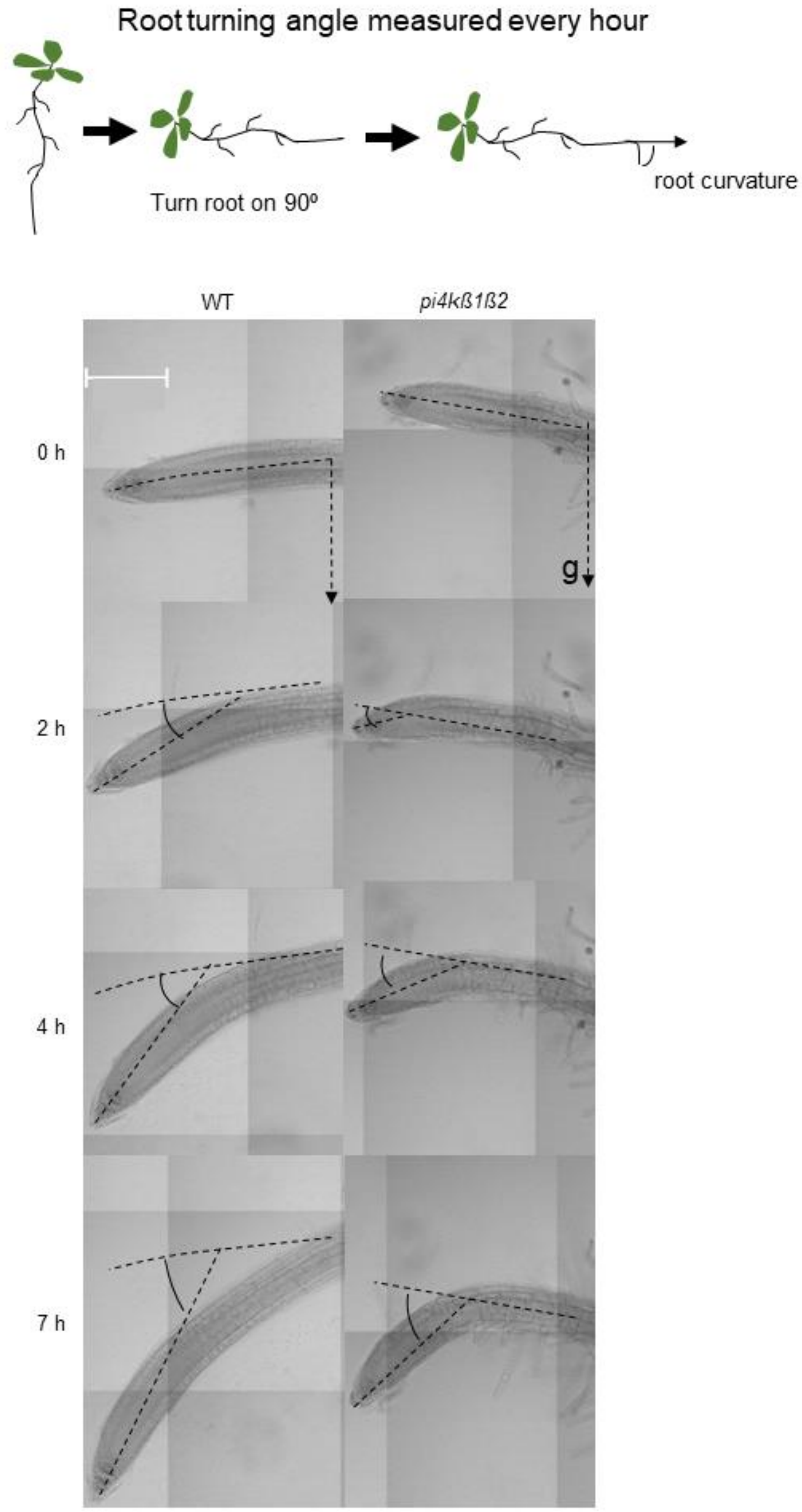

Supplementary fig. S6 Enrichment in GO categories in the sets of genes induced (230 genes) or repressed (264 genes) in *pi4kß1ß2* versus WT (Biological processes, molecular functions, cellular components). To focus on the most significant changes, we applied a log2- fold-change filter. Genes with the differential expression higher or lower than 1.5 were classified using the Classification SuperViewer Tool developed by (Provart and Zhu, 2003). The classification source was set to Gene Ontology categories as defined by (Ashburner et al., 2000). The frequency of a category, normalised to that in the whole Arabidopsis set. The mean and standard deviation for 100 bootstraps of our input set were calculated to provide some idea about over- or under-representation reliability.

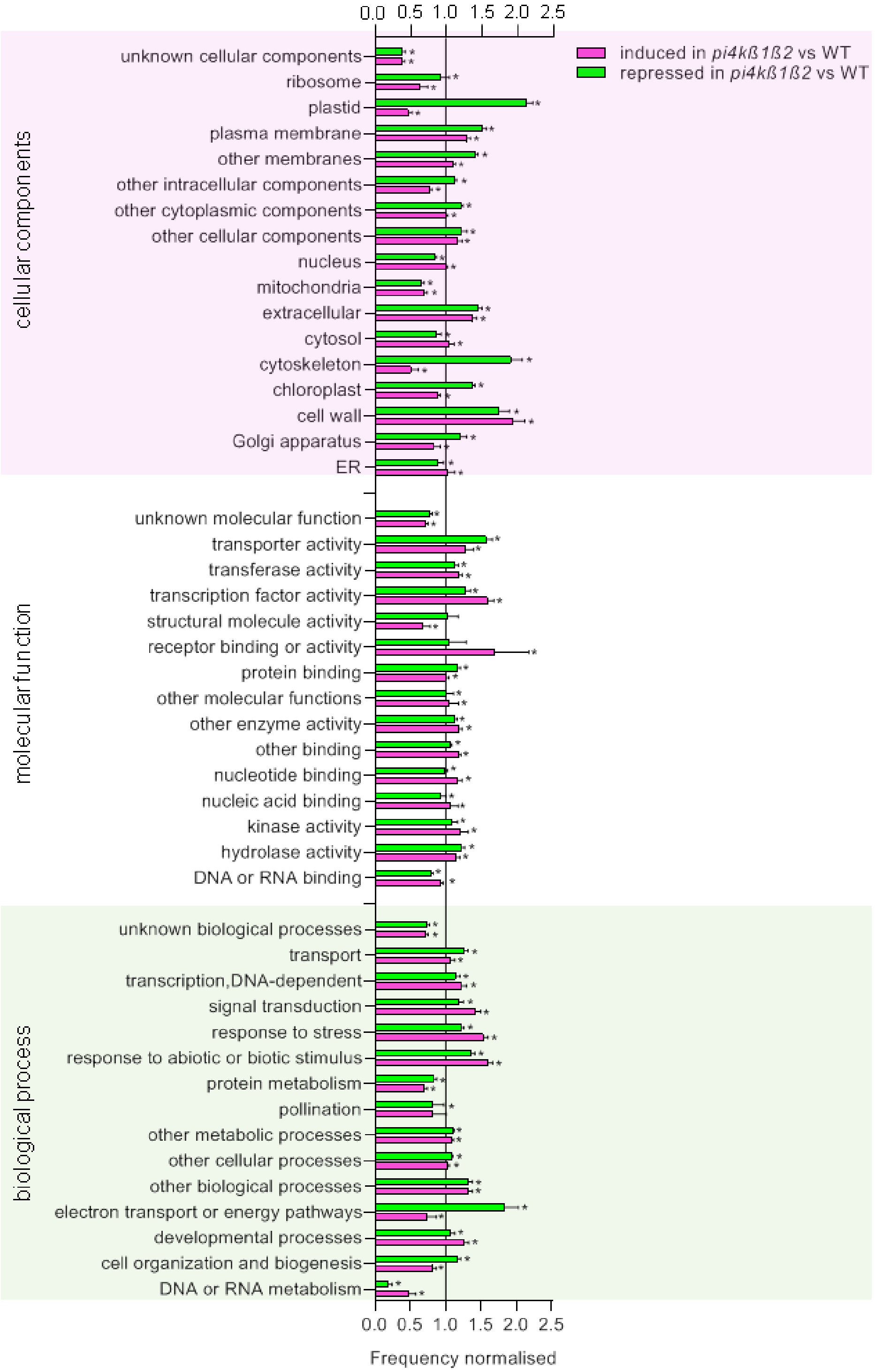

Supplementary fig. S7 Transcript levels of selected up- and down-regulated genes in *pi4kß1ß2* plants versus the WT. **a**, selected genes, with the log2foldChange as detected in the NGS experiment; **b**, transcript levels of the selected genes as measured by qPCR; Root samples were collected from 11-day-old seedlings of *A. thaliana* Col-0 (WT) and the *pi4kß1ß2* mutant. Values were normalized to the WT. *TIP41* was used as a reference gene. Data represents mean + SEM, one-way ANOVA with Tukey-HSD post-hoc test, n=3.

a

id	Annotation	log2FoldChange	adjusted.PValue
AT1G04240 *(SHY2)*	AUX/IAA transcriptional regulator family protein	-1.45	2.47E-26
AT5G23060 *(CAS)*	calcium sensing receptor	-1.24	9.13E-13
AT1G44575 *(NPQ)*	Chlorophyll A-B binding family protein	-1.15	4.40E-16
AT1G75690 *(LQY)*	DnaJ/Hsp40 cysteine-rich domain superfamily protein	-1.71	1.47E-14
AT1G72610 *(GER)*	germin-like protein 1	-1.84	2.10E-13
AT2G40340 *(DREB2C)*	Integrase-type DNA-binding superfamily protein	0.75	1.48E-02

b

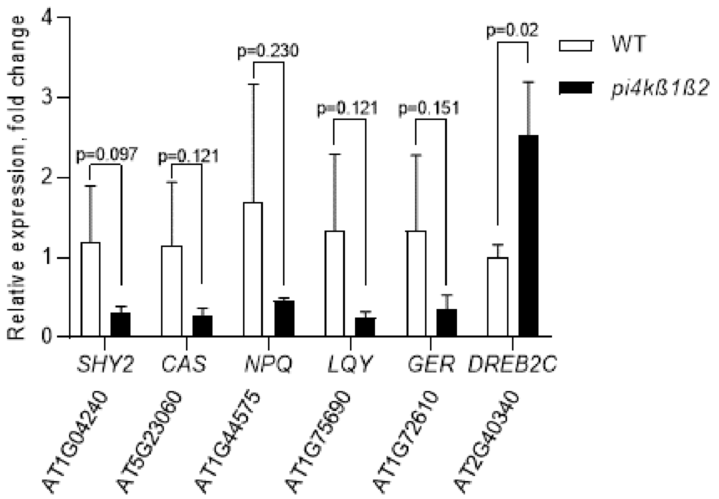

Supplementary fig. S8 The 20 most induced (a) and 20 most repressed (b) genes for *pi4kß1ß2* mutant roots versus WT roots.

a

id	Annotation	log2FoldChange	adjusted.PValue
AT5G19880	Peroxidase superfamily protein	3.63	2.20E-37
AT2G29350	senescence-associated gene 13	5.43	9.30E-40
AT1G26390	FAD-binding Berberine family protein	4.89	1.54E-40
AT1G26410	FAD-binding Berberine family protein	4.84	3.69E-51
AT2G30660	ATP-dependent caseinolytic (Clp) protease/crotonase family protein	4.66	1.22E-12
AT4G28420	Tyrosine transaminase family protein	4.46	6.99E-20
AT2G30770	cytochrome P450, family 71, subfamily A, polypeptide 13	4.45	3.51E-25
AT2G30750	cytochrome P450, family 71, subfamily A, polypeptide 12	4.39	4.24E-28
AT4G31970	cytochrome P450, family 82, subfamily C, polypeptide 2	4.27	1.45E-11
AT1G52120	Mannose-binding lectin superfamily protein	4.23	8.68E-17
AT5G13320	Auxin-responsive GH3 family protein	4.13	3.80E-46
AT1G08080	alpha carbonic anhydrase 7	4.12	3.04E-11
AT1G52130	Mannose-binding lectin superfamily protein	4.11	1.22E-21
AT1G26240	Proline-rich extensin-like family protein	4.11	9.37E-32
AT1G01680	plant U-box 54	4.07	7.85E-11
AT3G47480	Calcium-binding EF-hand family protein	4.03	6.13E-31
AT4G15370	baruol synthase 1	3.92	4.60E-30
AT5G05340	Peroxidase superfamily protein	3.85	3.25E-57
AT3G60470	Plant protein of unknown function (DUF247)	3.68	1.57E-19
AT4G15100	serine carboxypeptidase-like 30	3.67	3.10E-25

b

id	Annotation	log2FoldChange	adjusted.PValue
AT1G61130	serine carboxypeptidase-like 32	-3.34	9.02E-26
AT3G08900	reversibly glycosylated polypeptide 3	-3.41	1.08E-06
AT3G13784	cell wall invertase 5	-3.47	4.88E-13
AT3G46400	Leucine-rich repeat protein kinase family protein	-3.57	2.00E-16
AT4G23496	SPIRAL1-like5	-3.58	2.70E-51
AT1G78440	Arabidopsis thaliana gibberellin 2-oxidase 1	-3.64	1.03E-14
AT2G42250	cytochrome P450, family 712, subfamily A, polypeptide 1	-3.68	2.63E-75
AT5G06900	cytochrome P450, family 93, subfamily D, polypeptide 1	-3.76	2.63E-12
AT1G78450	SOUL heme-binding family protein	-3.78	2.83E-12
AT1G52820	2-oxoglutarate (2OG) and Fe(II)-dependent oxygenase superfamily protein	-4.03	8.65E-168
AT3G62740	beta glucosidase 7	-4.25	9.48E-33
AT4G01890	Pectin lyase-like superfamily protein	-4.35	1.02E-26
AT5G06905	cytochrome P450, family 712, subfamily A, polypeptide 2	-4.38	2.51E-13
AT2G33810	squamosa promoter binding protein-like 3	-4.48	3.93E-13
AT3G30260	AGAMOUS-like 79	-4.70	8.13E-20
AT1G52790	2-oxoglutarate (2OG) and Fe(II)-dependent oxygenase superfamily protein	-5.03	2.50E-10
AT2G01280	Cyclin/Brf1-like TBP-binding protein	-5.16	3.63E-11
AT3G52970	cytochrome P450, family 76, subfamily G, polypeptide 1	-5.81	8.86E-26
AT1G53480	mto 1 responding down 1	-5.96	4.04E-47
AT2G24000	serine carboxypeptidase-like 22	-7.38	7.40E-16

Supplementary fig. S9 3D reconstruction root immunostaining against PIN2. Merged 3D reconstruction of pictures taken along the z-axis of the fluorescent channel to track distribution of PIN2 along the plasma membrane in **a**, WT and **b**, *pi4kß1ß2* background. Scale bar 10 mm. Color lines represent axes in 3D reconstruction (X-red, Y-green, Z-Blue).

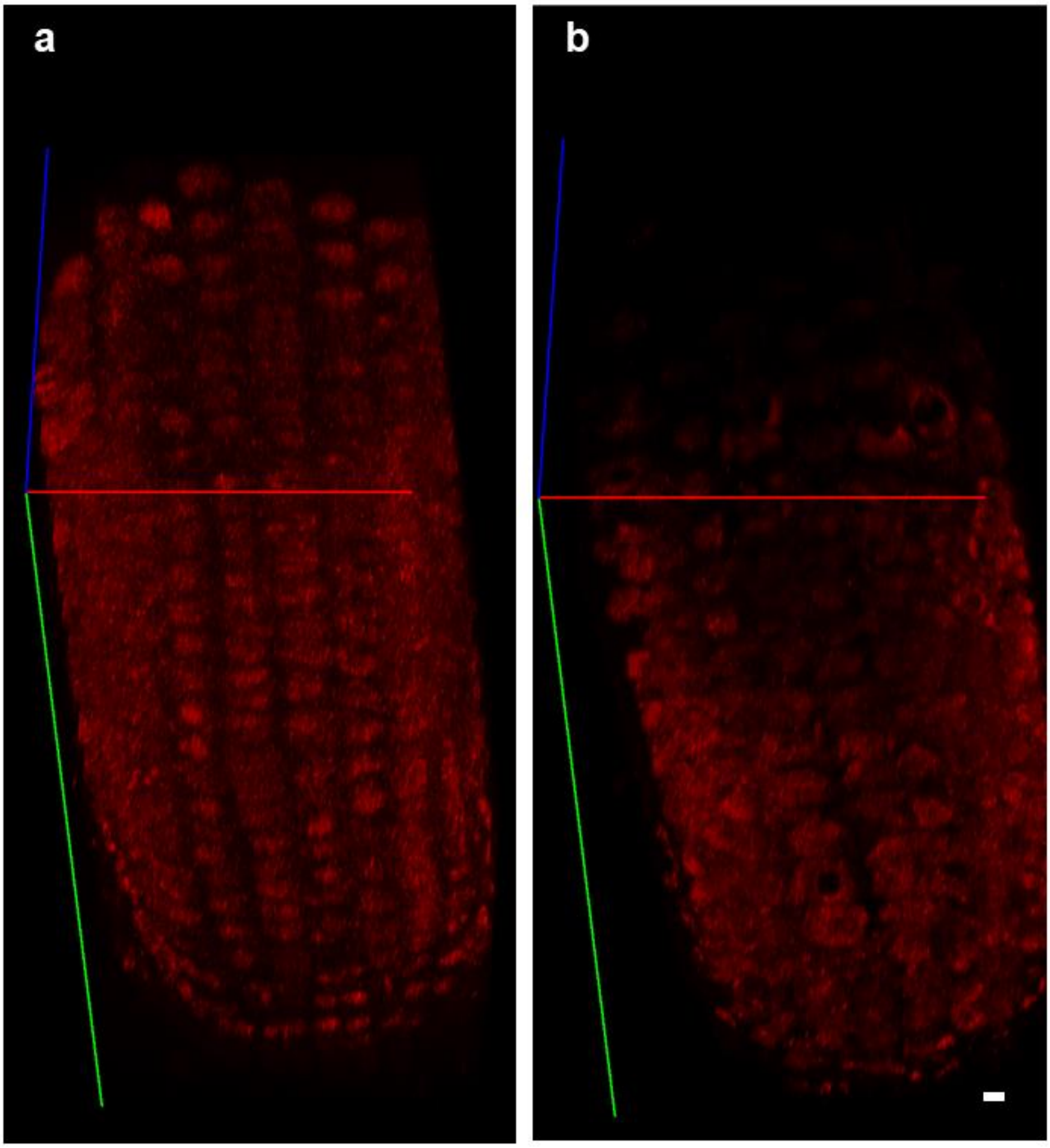

Supplementary fig. S10 3D reconstruction root epidermis cell transition zone. Merged 3D reconstruction of pictures taken along the z-axis of the brightfield and fluorescent channel of PIN2:GFP distribution along the plasma membrane and vacuole morphology in **a**, WT and **b**, *pi4kß1ß2* background. Scale bar 10 mm.

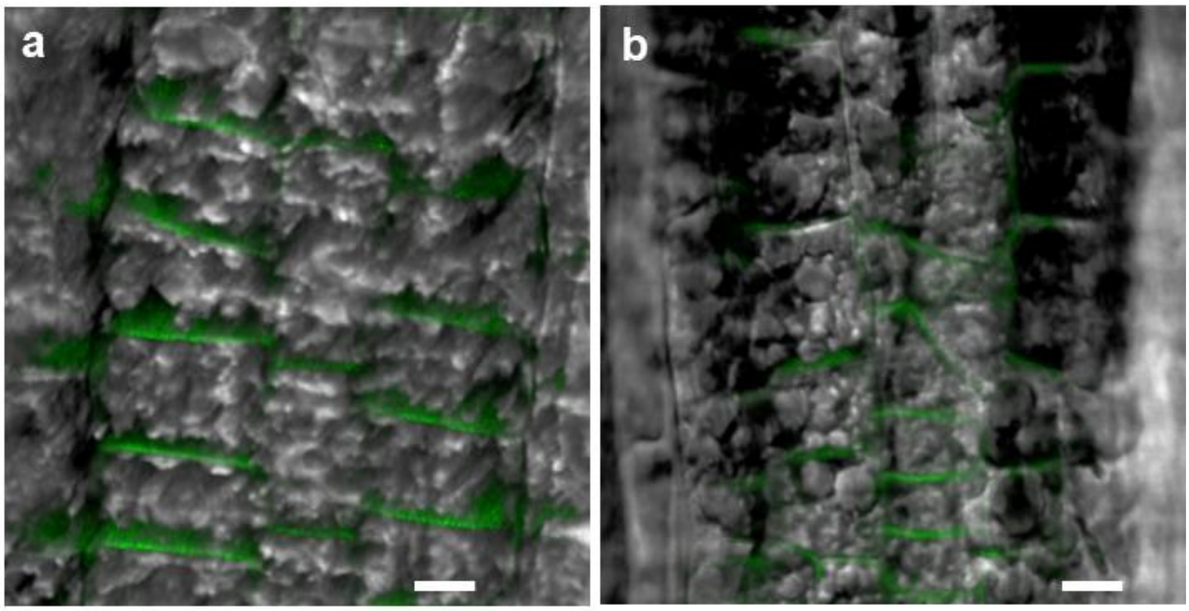

Supplementary fig. S11 Salicylic acid content of WT and *pi4kß1ß2* roots. Whole root systems (50-100 mg FW per sample) were harvested from 7-day-old vertical grown seedlings, n=6.

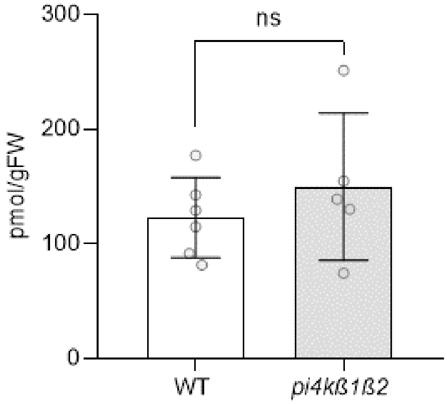

Supplement: Supplementary file 11 — Supplementary Information 16. [file 41598_2022_10458_MOESM11_ESM.pdf]
